# How to Build a Functional Connectomic Biomarker for Mild Cognitive Impairment From Source Reconstructed MEG Resting-State Activity: The Combination of ROI Representation and Connectivity Estimator Matters

**DOI:** 10.3389/fnins.2018.00306

**Published:** 2018-06-01

**Authors:** Stavros I. Dimitriadis, María E. López, Ricardo Bruña, Pablo Cuesta, Alberto Marcos, Fernando Maestú, Ernesto Pereda

**Affiliations:** ^1^Division of Psychological Medicine and Clinical Neurosciences, School of Medicine, Cardiff University, Cardiff, United Kingdom; ^2^Cardiff University Brain Research Imaging Centre, School of Psychology, Cardiff University, Cardiff, United Kingdom; ^3^School of Psychology, Cardiff University, Cardiff, United Kingdom; ^4^Neuroinformatics Group, Cardiff University Brain Research Imaging Centre, School of Psychology, Cardiff University, Cardiff, United Kingdom; ^5^Neuroscience and Mental Health Research Institute, Cardiff University, Cardiff, United Kingdom; ^6^Department of Basic Psychology II, Complutense University of Madrid, Madrid, Spain; ^7^Laboratory of Cognitive and Computational Neuroscience, Center for Biomedical Technology, Madrid, Spain; ^8^Networking Research Center on Bioengineering, Biomaterials and Nanomedicine (CIBER-BBN), Zaragoza, Spain; ^9^Electrical Engineering and Bioengineering Group, Department of Industrial Engineering and IUNE, Universidad de La Laguna, Tenerife, Spain; ^10^Department of Neurology, San Carlos University Hospital, Madrid, Spain

**Keywords:** connectomic biomarker, magnetoencephalography, mild cognitive impairment, virtual source activity, connectome data analysis, multiplexity, cross-frequency-coupling, intrinsic coupling modes

## Abstract

Our work aimed to demonstrate the combination of machine learning and graph theory for the designing of a connectomic biomarker for mild cognitive impairment (MCI) subjects using eyes-closed neuromagnetic recordings. The whole analysis based on source-reconstructed neuromagnetic activity. As ROI representation, we employed the principal component analysis (PCA) and centroid approaches. As representative bi-variate connectivity estimators for the estimation of intra and cross-frequency interactions, we adopted the phase locking value (PLV), the imaginary part (iPLV) and the correlation of the envelope (CorrEnv). Both intra and cross-frequency interactions (CFC) have been estimated with the three connectivity estimators within the seven frequency bands (intra-frequency) and in pairs (CFC), correspondingly. We demonstrated how different versions of functional connectivity graphs single-layer (SL-FCG) and multi-layer (ML-FCG) can give us a different view of the functional interactions across the brain areas. Finally, we applied machine learning techniques with main scope to build a reliable connectomic biomarker by analyzing both SL-FCG and ML-FCG in two different options: as a whole unit using a tensorial extraction algorithm and as single pair-wise coupling estimations. We concluded that edge-weighed feature selection strategy outperformed the tensorial treatment of SL-FCG and ML-FCG. The highest classification performance was obtained with the centroid ROI representation and edge-weighted analysis of the SL-FCG reaching the 98% for the CorrEnv in α_1_:α_2_ and 94% for the iPLV in α_2_. Classification performance based on the multi-layer participation coefficient, a multiplexity index reached 52% for iPLV and 52% for CorrEnv. Selected functional connections that build the multivariate connectomic biomarker in the edge-weighted scenario are located in default-mode, fronto-parietal, and cingulo-opercular network. Our analysis supports the notion of analyzing FCG simultaneously in intra and cross-frequency whole brain interactions with various connectivity estimators in beamformed recordings.

## Introduction

Mild cognitive impairment (MCI) is a brain disease with both anatomical and functional alterations and first episodes of cognitive impairments complementary to other factors like education and age (Petersen et al., 1999). MCI can be seen as a transitional stage between normal aging and dementia where a subject can continue his/her daily activities. There are clear evidences that individuals that are diagnosed as MCI have a high risk to develop dementia in the next 2–5 years compared to age-matched population with non-MCI diagnosis (AD; Shah et al., 2000; Farias et al., 2005). Specifically, MCI subjects with accumulation of intracellular Tau, medial temporal atrophy and amyloid deposition are classified clinically as predementia phase of AD (Albert et al., 2011). All of these pathological biomarkers cause synaptic disruptions (Braak and Braak, 1991). In the literature, quite often AD has been named as a dis-connection syndrome in cellular and macroscale level. This is a wrong term that makes a lot of neuroscientists in any scale of research around AD to believe that some brain areas are completely isolated from the rest of the brain network during AD. Practically, instead of disconnection syndrome one can use the term “functional disruption syndrome” (Delbeuck et al., 2003; Arendt, 2009; Takahashi et al., 2010; Koelewijn et al., 2017). Alterations of anatomical and functional alterations have been reported during the MCI pre-AD stage (Pijnenburg et al., 2004; Koenig et al., 2005; Buldú et al., 2011; Wang et al., 2013).

For a better understanding of how the various anatomical brain areas communicate, functional connectivity (FC) should be explored (Friston, 2011). Many resting-state studies using electroencephalography (EEG) and magnetoencephalography (MEG) have revealed a decrease in FC especially in α and β frequencies in MCI patients compared to healthy controls (Moretti et al., 2008; Gómez et al., 2009; López et al., 2014a; Cuesta et al., 2015). This functional pattern is close to the one reported for AD patients (Stam and van Dijk, 2002; Jeong, 2004; Stam et al., 2006; Koelewijn et al., 2017), although in a few studies an increased functional pattern have been revealed in posterior brain areas (Stam et al., 2006; Alonso et al., 2011).

Deviations of FC from normal have been revealed in MCI within the default mode network (DMN) with similar disruptions in anatomical connections (Garcés et al., 2014; Pineda-Pardo et al., 2014). Only in a few resting-state neuromagnetic studies where different MCI groups were compared, a specific hyper-synchronization pattern was untangled in both α and β frequency bands in MCI subjects that finally transited to AD (López et al., 2014b). Similar results have been presented to subjects with an abnormal concentration of phospho-tau protein in the cerebrospinal fluid (CSF; Canuet et al., 2015). In a recent multi-center study, the profile of hyper-synchronization was proved valuable to build a connectomic biomarker with high classification performance of MCI versus healthy controls (Maestú et al., 2015).

Most studies that attempted to define a reliable connectomic biomarker for the detection of MCI using EEG/MEG FC analyzed functional interactions between brain activities within the same frequency band (intra-frequency interactions). Recently, we designed a novel biomarker based on an EEG-based auditory oddball paradigm building a multi-parametric biomarker based on Pz activity and dynamic reconfiguration of cross-frequency coupling (CFC) (Dimitriadis et al., 2015a). CFC is an integrated mechanism that increases the timing of synchronization between distant brain areas oscillating on slow and fast frequencies and there are many neuroscientific evidences that support its existence in both resting-state and cognition (Canolty and Knight, 2010; Palva and Palva, 2011; Buzsáki and Watson, 2012; Jirsa and Müller, 2013; Dimitriadis et al., 2015b, 2016b). In a recent study, we demonstrated alterations of specific cross-frequency coupling patterns due a mnemonic strategy training protocol in elderly at risk of AD (Dimitriadis et al., 2016d). We revealed alterations of CFC in dyslexia (Dimitriadis et al., 2016a) and in mild traumatic brain injury (Antonakakis et al., 2016, 2017a) using neuromagnetic recordings at resting-state. For that reason, CFC should be explored in conjunction with intra-frequency coupling in a single integrated FC graph (SL-FCG; Dimitriadis et al., 2015b, 2016b; Antonakakis et al., 2016, 2017a; Dimitriadis, 2016a; Dimitriadis and Salis, 2017) and/or in a multi-layer FCG (ML-FCG; Brookes et al., 2016).

A connectomic biomarker can be designed by adopting different strategies focusing on graph theory and network neuroscience. The simplest way is to apply a supervised feature selection algorithm using every possible pair of connections as a single feature and using a number of edges' weights as a multiparametric biomarker to evaluate the performance via a cross-validation procedure such as leave-one-out cross-validation; (LOOCV) or k-fold CV (Maestú et al., 2015). This approach can be used on every intra and cross-frequency version of the FCG and on the multi-layer FCG. Alternatively, the FCG can be treated as a 2D tensor. In that case, proper techniques should be adopted tailored to tensorial learning and classification commonly used in computer vision and image processing (Dimitriadis et al., 2015b, 2016d; Antonakakis et al., 2016, 2017a). In the case of the tensorial treatment of a FCG, in both SL-FCG and ML-FCG formats, two different approaches can be used. The fully-weighted versions of the FCGs and the topological filtered versions using a data-driven technique. Here, we adopted our novel data-driven topological filtering technique called orthogonal minimal spanning trees (OMST; Dimitriadis et al., 2017a,c).

Source reconstruction of neuromagnetic recordings demands the selection of an atlas. The majority of the studies employed AAL-90 atlas in order to define functional ROIs. However, there is no study in the literature to report how the representative ROI time series could affect functional brain networks. Practically, a number of voxel time series constrained by the boundaries of atlas template should be proper analyzed in order to get the characteristic time series per ROI. Here, we tested the most characteristic, the PCA and the centroid.

In this work, we explored alternative ways that will improve the discrimination of MCI from age-matched controls using MEG activity in the source domain. To demonstrate the whole analysis, we estimated static functional brain networks from neuromagnetic resting-state recordings (eyes-open). The strength of functional interactions between two brain sources was estimated using the imaginary part of phase locking value (Dimitriadis et al., 2015a, 2016a,b,c,d; Antonakakis et al., 2016, 2017a; Bruna et al., 2017), the original phase locking value and the amplitude envelope correlation (CorrEnv) (Brookes et al., 2011a,b) as representative estimators of frequency-resolved FC for the phase and the amplitude, respectively. Both estimators have been used to quantify the coupling between every possible pair of sources with the same frequency content (intra-frequency interactions) and CFC (Fitzgerald et al., 2013). Here, we adopted the most characteristic connectivity estimators for both amplitude and phase domain.

The last year, neuroscience community reported the notion of multi-layer functional brain networks as a new tool in network brain science. First preliminary results reported loss of multiplexity in Alzheimer's disease (Guillon et al., 2017) and particularly in hippocampus and posterior hubs (Yu M. et al., 2017). However, in their analysis, they constructed the multi-layer functional brain networks only with intra-frequency coupling functional brain networks. Here, we will test the performance of multi-layer participation coefficient in MCI subjects including also cross-frequency layers. It is important to underline that statistical difference between MPC values doesn't mean a high classification performance while the classification performance in AD is of no clinical value. Our goal must be to design neuroinformatic tools sensitive to prodromal AD stages like MCI.

Significantly, there are two basic functional brain networks that increase their activity during the performance of many cognitive tasks, the fronto-parietal network (FPN) and the cingulo-opercular network (CON) (Dosenbach et al., 2006). In many cases the within-network functional connectivity strength can predict the cognitive performance (Kelly et al., 2008; Song et al., 2008) implicating them as part of the core brain system for task controlling that implies global cognition. Unfolding the key role of both functional brain networks, it has been proved that abnormalities in the control supported by these two networks can lead to mental illness (Cole et al., 2014). We already know that the pathology of AD is distributed in high—order cognitive functions including episodic memory retrieval. Two main networks have been revealed to be linked to episodic memory retrieval, the fronto-parietal and the cingulo-opercular (Dhanjal and Wise, 2014). Complementary, medial temporal lobe activity has been linked to cognitive decline in MCI (Maestú et al., 2006) while incidental emotional memory based on emotional pictures triggers parahippocampal brain areas in a less extent in MCI compared to healthy controls (Parra et al., 2013). DMN is expected also to be disrupted in MCI (Garcés et al., 2014). We hypothesize that FPN, DMN, and CON will contribute to the multivariate connectomic biomarker for MCI based on neuromagnetic recordings at resting-state.

Finally, we will show the benefits of constructing a single-graph by untangling the dominant intrinsic coupling mode per pair of EEG/MEG sensors/sources (FCGDICM; Antonakakis et al., 2016, 2017a; Dimitriadis, 2016a,b; Dimitriadis and Salis, 2017). The same procedure will be followed here for both estimators.

The main goal of this study is to explore the performance of different analytic strategies of single-layer or multi-layer representations of functional brain networks. Additionally, we aim to report how the selection of ROI representation and the connectivity estimator could alter the performance of a functional connectomic biomarker. The analysis focuses on whole-brain static functional brain networks with both intra and cross-frequency interactions employing representative connectivity estimators for both amplitude and phase domain. Our analytics underline the need of further exploration of the preprocessing pipeline for neuromagnetic recordings tailored to the definition of a reliable functional connectomic biomarker for mild cognitive impairment.

The aforementioned different choices in every step of the analysis (from the extraction of the source time series till the construction of a static FCG) are demonstrated using a representative set of healthy controls and MCI subjects. In Materials and Methods section, we described the data acquisition, the beamforming analysis to reconstruct the sources, the MEG analysis, the construction of the various versions of a FCG and the alternative classification approaches. The Results section is devoted to describe the results including classification performance, sensitivity, and specificity of the alternative choices. Finally, the Discussion section includes the discussion of the current research results with future extensions.

## Materials and methods

### Subjects and ethics statement

Data was obtained for 24 subjects diagnosed with mild cognitive impairment (MCI) (11 males, age 72.77 ± 3.31 years old, mean ± SD) and 30 healthy controls (13 males, age 72.37 ± 2.63 years old). The MCI group and the control group were recruited from the Hospital Clínico Universitario San Carlos (Madrid). All subjects were right handed and native Spanish speakers (Oldfield, 1971). Table 1 summarizes the demographic features and mean hippocampal volumes of the subjects in both groups.

**Table 1:** Mean ± standard deviation of the demographic characteristics of controls and MCIs.

	**Number of subjects**	**Gender (M/F)**	**Age**	**MMSE**	**LH ICV**	**RH ICV**
Control	30	13/17	72.37 ± 2.63	29.13 ± 0.94	0.0026 ± 0.0003	0.0026 ± 0.0003
MCI	24	12/11	72.67 ± 3.31	26.43 ± 3.22	0.0021 ± 0.0003	0.0022 ± 0.0004

*M, males; F, females; MMSE, Mini-Mental State Examination; LH ICV, Left hippocampus normalized by total intracranial volume (ICV); RH ICV, Right hippocampus normalized by ICV*.

To explore their cognitive and functional status, all participants were screened by means of a variety of standardized diagnostic instruments and underwent an extensive cognitive assessment, as described in López et al. (2016).

The main criteria for the diagnosis of MCI according to the National Institute of Aging – Alzheimer Association (NIA-AA) criteria (Albert et al., 2011; López et al., 2014a,b) are:

1. self- or informant-reported cognitive complaint;
2. objective evidence of cognitive impairment;
3. preserved independence in functional abilities and
4. not fulfilling the criteria for dementia (McKhann et al., 2011; López et al., 2014a,b). All of them were categorized as “MCI due to AD intermediate likelihood.” Besides, they all presented hippocampal atrophy (see Table 1), which was measured by magnetic resonance (MRI). According to their cognitive profile, they were classified as amnestic subtype (Petersen et al., 1999).

Methods were carried out in accordance with the approved guidelines and general research practice. The study was approved by the Hospital Clínico Universitario San Carlos (Madrid) ethics committee. All participants or their guardians filled and signed a written informed consent prior to participation.

### MEG acquisition and preprocessing

Biomagnetic data was acquired using a 306-channel Elekta Vectorview system (Elekta AB, Stockholm, Sweden) placed inside a magnetically shielded room (VacuumSchmelze GmbH, Hanau, Germany) located at the Laboratory of Cognitive and Computational Neuroscience (Madrid, Spain). Signal was recorded while the subjects were awake, sitting comfortably and with their eyes open, while looking at a white fixation cross projected on a screen.

Prior to the MEG recording, two electrodes were placed above and below the left eye, in a bipolar montage, in order to acquire electro-oculographic activity. Four head position indicator (HPI) coils were placed in the head of the subject, two in the forehead and two in the mastoids, in order to online estimate the head position. Position of the three fiducial points, along with the HPI coils and over 200 evenly spaced points of the head shape of the subject, were acquired using a three-dimensional Fastrack digitizer (Polhemus, Colchester, Vermont). The HPI coils were fed during the whole acquisition, allowing for offline estimation of the head position.

Four minutes of resting state activity were acquired from each subject. Data was online filtered between 0.1 and 330 Hz, and digitized using a sampling rate of 1,000 Hz. After the acquisition, recordings were offline processed using the spatiotemporal extension of the signal separation algorithm (tSSS) (Taulu et al., 2004). Parameters for the tSSS were a window length of 10 s and a correlation threshold of 0.9. This algorithm removes the signals whose origin is estimated outside the MEG helmet, while keeping intact the signals coming from inside the head. In addition, the continuous HPI acquisition, combined with the tSSS algorithm, allowed for the continuous movement compensation. As result, the signal used in the next steps comes from a set of virtual sensors whose position remains static respect to the head of the subject. Those subjects whose movement along the recording was larger than 25 mm were discarded, following the recommendations of the manufacturer.

Data was examined using the automatic artifact detection of FieldTrip toolbox (Oostenveld et al., 2011), looking for ocular, muscular, and jump artifacts. The detected artifacts were confirmed by a MEG expert, in order to correct both false positives and negatives. Muscular and jump artifacts were marked as destructive artifacts, and segments containing them were completely discarded. On the remaining segments, a blind source separation algorithm based in second order statistics (SOBI) was used to obtain statistically independent components. SOBI components were labeled as oculographic, cardiographic, noisy components or real data. Artifact-related components were eliminated, and segments containing persistent oculographic artifacts were removed. Last, data was segmented in 4-s epochs of artifact-free data. Subjects with <20 epochs were discarded from the analysis, due to a low signal to noise ratio.

### MRI acquisition and processing

A T1-weighted MRI was acquired for each subject in a General Electric 1.5 T scanner, using a high-resolution antenna and a PURE filter (Fast Spoiled Gradient Echo sequence, TR = 11.2 ms, TE = 4.2 ms, TI = 450 ms; flip angle of 12°; slice thickness of 1 mm; FOV of 25 cm, 256 × 256 matrix). MRI images were segmented in gray matter (GM), white matter (WM), cerebrospinal fluid (CSF) bone and soft tissue using SPM version 12 (Ashburner and Friston, 2001). A binary mask for the brain was generated using those voxels whose combined probability of WM, GM, and CSF were >0.5. Last, a mesh surface was generated from the defined mask using FieldTrip.

### Source reconstruction

A volumetric grid was generated for the MNI template, using a homogenous separation of 1 cm in each direction, with one source placed in (0, 0, 0) in MNI coordinates. Only sources inside the brain surface (as defined in the previous section) were taking in account, resulting in a source model with 2,459 sources, each consisting in three perpendicular dipoles. Each source was labeled according to the automatic anatomical labeling (AAL) atlas (Tzourio-Mazoyer et al., 2002). The final number of sources considered, as only cortical ones were used, was 1,467.

The defined grid was transformed to subject space using the original T1 image. Both the grid and the brain surface were manually realigned to Neuromag coordinate system using the three fiducials and the head shape as guides. A lead field was calculated using a realistic single shell head (Nolte, 2003) as forward model. The source reconstruction was performed using a Linearly Constrained Minimum Variance (LCMV) beamformer (Van Veen et al., 1997) for broadband. The resulting spatial filters were projected over the maximal radiation direction, getting only one filter per source. Source-space time series were reconstructed and grouped according to the atlas, obtaining one representative time series for area using (1) the PCA of all the sources in the area or (2) the source closest to the centroid of the area (CENT).

**Figure 1:**
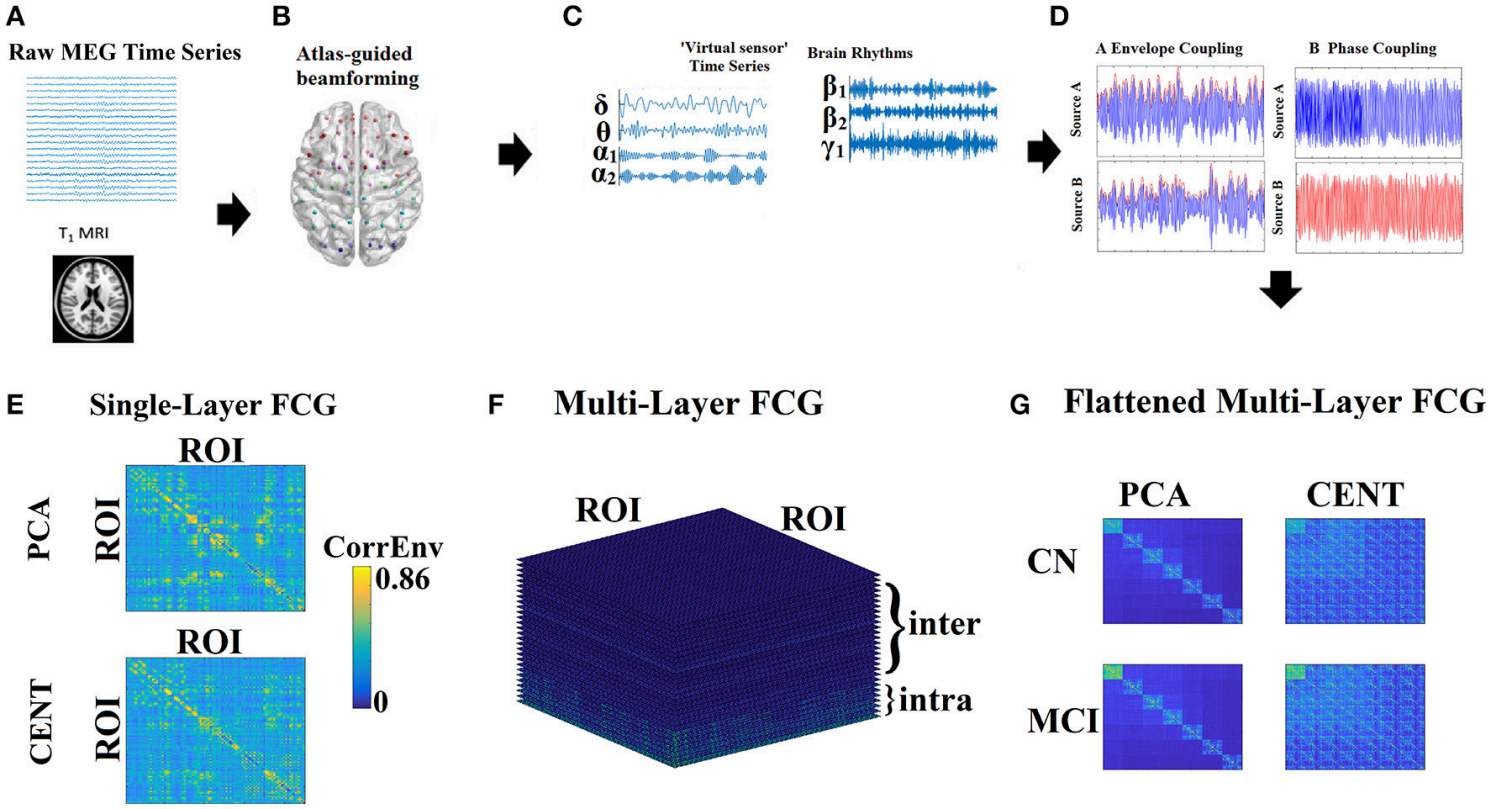
From MEG recordings to single-layer and multi-layer FCG. **(A)** Raw MEG time series recordings and T1 MRI image. **(B)** Atlas-guided beamforming (here AAL-90 template). **(C)** Virtual sensors time series for each brain rhythm. **(D)** Estimation of the functional connectivity with the CorrEnv based on the Hilbert envelope and the iPLV based on the Hilbert phase time series. **(E)** Single-layer FCG: an example from a healthy control subject in the δ frequency band demonstrating both types of ROI representation for contrast. **(F)** Multi-Layer FCG: in our study, we used 7 intra-frequency intrinsic coupling modes and 21 inter-frequency coupling modes, leading to 28 in total. **(G)** Flattened Multi-Layer FCG: The dimension of the flattened Multi-Layer FCG equals {Coupling Modes × ROI} × {Coupling Modes × ROI}, where in the main diagonal FCG of intra-frequency coupling modes are inserted while in the off-diagonal FCG of inter-frequency coupling modes are encapsulated. Where Coupling Modes = 28 intra and inter-frequency FCG. PCA, principal component analysis; CENT, centroid; CN, control; MCI, mild cognitive impairment.

The whole process from data collection to the extraction of the filtered time series is briefly depicted in Figures 1A–C.

### MEG analysis

We selected, per each subject, multiple artifact free trials of 6 s (6,000 samples) after careful visual inspection, giving 32–44 epochs for further analysis. Time-series of neuronal activation were computed for the seven frequency bands: δ (0.5–4 Hz), θ (4–8 Hz), α_1_ (8–10 Hz), α_2_ (10–13 Hz), β_1_ (13–20 Hz), β_2_ (20–30), γ_1_ (30–45 Hz) using a third order Butterworth filter with zero-phase using *filtfilt.m* function from MATLAB (Figure 1C).

### Functional connectivity

#### Imaginary part of phase locking value (iPLV)

Phase synchrony between two source time series within a particular frequency band was assessed via the estimates of the instantaneous phase of the signal. In both task and resting-state literature, these measures are computed within each trial and taking average values across all epochs (Lachaux et al., 1999).

The complex analytic representations of each signal z(t) is derived via the Hilbert transform (HT[.]):

(1) $$\begin{array}{r} \text{z}\left(\text{t}\right)=\text{HT}\left[\text{x}\left(\text{t}\right)\right]=|\text{z}\left(\text{t}\right)|{\text{e}}^{\text{iϕ}F\left(t\right)}=\text{ALF}\left(\text{t}\right)e^{\text{iϕ}F\left(t\right)} \end{array}$$

Phase consistency between the two signals is measured by means of both the original definition (Lachaux et al., 1999; Mormann et al., 2000; Figure 1D) and the imaginary part of PLV (iPLV), as synchronization indexes to quantify the strength of PAC.

The original *PLV* is defined as follows:

(2) $$\begin{array}{r} PLV=\frac{1}{T}|\overset{T}{\underset{t=1}{\sum }}e^{i\left(\varphi_{X}\left(t\right)-\varphi_{Y}\left(t\right)\right)}| \end{array}$$

and the imaginary part of *PLV* as follows:

(3) $$\begin{array}{r} ImPLV=\frac{1}{T}|Im\overset{T}{\underset{t=1}{\sum }}e^{i\left(\varphi_{X}\left(t\right)-\varphi_{Y}\left(t\right)\right)}| \end{array}$$

The imaginary part of PLV is less susceptible to volume conduction effects in assessing CFC interactions and was used in all subsequent analyses. iPLV is less affected by volume conduction, it could be sensitive in some cases to alterations in the angle between two time series, which do not necessarily is related to a PLV change. iPLV is only sensitive to non-zero-phase lags and is thus resistant to instantaneous self-interactions associated with volume conductance (Nolte et al., 2004).

iPLV has been used by our group to quantify both intra and cross-frequency interactions namely the phase-to-amplitude coupling (PAC) between the phase of the slower rhythm and the phase of the slower rhythm within the high frequency amplitude (Dimitriadis et al., 2015a, 2016a,b,c,d; Bruna et al., 2017). See below the basic preprocessing steps for the estimation of PAC.

Recent studies demonstrated that imaginary part of *PLV* (*iPLV*) can remove artificial interactions but it cannot eliminate spurious interactions if the true coupling has non-zero phase lag (Palva et al., 2018; Wang et al., 2018). They finally suggest that hyperedge bundling can significantly decreases graph noise by minimizing the false-positive to true-positive ratio (Wang et al., 2018).

A revisited study for phase-locking bivariate estimators demonstrated how corrected imaginary part of PLV (ciPLV) can give results robust to volume conduction and how functional connectivity graphs can be estimated faster (Bruna et al., 2017).

#### PAC estimation: the algorithmic steps

Let x(*t*), *t* = 1, 2, …, T is the virtual time series. Based on prefiltered versions of this signal, cross-frequency interactions will be estimated based on form of how the phase of low-frequency (LF) oscillations modulates the amplitude of high-frequency (HF) oscillations. Applying a narrowband filtering with a 3rd order zero-phase Butterworth filter, the two filtered signals x_LF_(*t*) and x_HF_(*t*) are first extracted. Then, applying Hilbert transform (HT[.]) to both filtered signals, the complex analytic representations z_LF_(*t*) and z_HF_(*t*) are derived

$$\begin{array}{rcl} {\text{Z}}_{\text{LF}}\left(t\right) & = & \text{HT}\left[{\text{X}}_{\text{LF}}\left(\text{t}\right)\right]=|{\text{Z}}_{\text{LF}}\left(\text{t}\right)|{\text{e}}^{\text{i}\phi_{\text{LF}}\left(t\right)}={\text{A}}_{\text{LF}}\left(\text{t}\right){\text{e}}^{\text{i}\phi_{\text{LF}}\left(t\right)} \end{array}$$

(4) $$\begin{array}{rcl} {\text{Z}}_{\text{HF}}\left(\text{t}\right) & = & \text{HT}\left[{\text{X}}_{\text{HF}}\left(\text{t}\right)\right]=|{\text{Z}}_{\text{HF}}\left(\text{t}\right)|{\text{e}}^{\text{i}\phi_{\text{HF}}\left(t\right)}={\text{A}}_{\text{HF}}\left(\text{t}\right){\text{e}}^{\text{i}\phi_{\text{HF}}\left(t\right)} \end{array}$$

The envelope A_HF_(*t*) signal of the higher frequency and the instantaneous phase φ(*t*) signal of the slower oscillation are extracted. Next, the envelope of the higher-frequency oscillations A_HF_(*t*) is band-pass filtered within the range of LF oscillations and the resulting signal undergoes an additional step of Hilbert transform so as to isolate its phase-dynamics component φ′(*t*),

(5) $$\begin{array}{r} z^{\prime }\left(t\right)=HT\left[{A_{HF}}_{,LF}\left(t\right)\right]=|z^{\prime }\left(t\right)|e^{i\phi_{HF}\left(t\right)}=|z^{\prime }\left(t\right)|e^{i\phi_{LF}\left(t\right)\to_{HF}\left(t\right)} \end{array}$$

Equation (5) reflects the modulation of HF-oscillations amplitude by the phase of the LF-oscillations. Finally, the corresponding time-series will be used to estimate PAC, by means of the imaginary part of phase-locking (or synchronization index) technique.

(6) $$\begin{array}{r} ImPLV_{LF\to HF=}\frac{1}{T}|Im\overset{T}{\underset{t=1}{\sum }}e^{i\left(\varphi_{HF}\left(t\right)-\varphi_{LF}\left(t\right)\right)}| \end{array}$$

Phase-locking value PLV ranges between 0 and 1, with higher values indicating stronger PAC interactions. Here, we estimated 21 CFC pairs based on the predefined number of frequencies.

Finally, 28 FCGs have been estimated per subject including the phase coupling of the sources within every frequency and 21 CFC pairs (for further details see Dimitriadis et al., 2016a).

#### Amplitude envelope correlation

We estimated the amplitude coupling between ROIs based on the correlations of the envelopes of signals within the same frequency (Brookes et al., 2012; Hipp et al., 2012) and with different frequency content (Fitzgerald et al., 2013). Here, 28 FCGs have been estimated per subject, including the AEC of the sources within every frequency and 21 CFC pairs (Figure 1D). Here, we used the non-orthogonalized version of AEC.

## Feature selection and cross-validation tailored to each FCG format

The different coupling modes (28 in total) of each FCG version can be analyzed as single-layer FCG (SL-FCG), each one with dimension 90 × 90 (Figure 1E), or as a multi-layer FCG (ML-FCG) with dimensions {7 × 90} × {7 × 90} (Figures 1F,G). In the main diagonal of this ML-FCG, blocks of intra-frequency couplings are tabulated, while in the off diagonal the CFC FCG are inserted.

### Feature selection and cross-validation tailored based on edge-weights

#### Feature selection

We adopted two different approaches for feature selection strategy. The first one refers to the selection of the edge weights as single features, while the second one is the tensorial treatment of FCG as a 2D matrix. For the former, we adopted the Minimum Redundancy Maximum Relevance (MCFS; Cai et al., 2010) feature selection, using mutual information as implemented in the feature selection toolbox (Roffo, 2016; Roffo and Melzi, 2017; Roffo et al., 2017). MCFS was used independently for each one of the 28 versions of SL-FCG and for the flattened ML-FCG. Feature selection strategy was followed at every fold in the CV phase and prior the training of the model, not prior to CV, in order to prevent overfitting the model and thereby improving the generalization of the proposed connectomic biomarker.

#### Classification scheme

For the functional edge feature selection approach, we employed support vector machines (SVM) with RBF kernel as a proper classifier. Here, we used two cross-validation schemes: LOOCV and the 5-fold. Feature selection strategy was followed at every fold on the training set in both CV schemes. Finally, we selected those features that were the most frequent across the folds. In most machine learning approaches, one selects a number of features or a percentage thereof at every fold for the feature selection algorithm and the number of features or its percentage that are more frequent selected across the folds. For example, we can select 100 features ranked with the feature selection algorithm and finally we can select the most 30 frequent across all the folds. This is an important step to first demonstrate the features and afterward to train the model for external blind classification. Here, we selected 15 features ranked with the feature selection algorithm and 15 most common features across the folds. Finally, sensitivity, specificity and classification performance will be reported in both validation schemes and FCG treatment.

### Feature selection and cross-validation tailored based on tensors

We proposed an alternative and more natural formulation of FCG, which is a 2D matrix. FCG can be seen and properly handled as tensors. Single-layer FCG (SL-FCG) is naturally a 90 × 90 2D matrix. Multi-layer FCG (ML-FCG) can be flattened to a 630 × 630 ({7 × 90} × {7 × 90}) 2D matrix. In both cases it is natural to deal with the matrices as 2D tensors (Figure 1E).

#### Feature extraction

**Figure 2:**
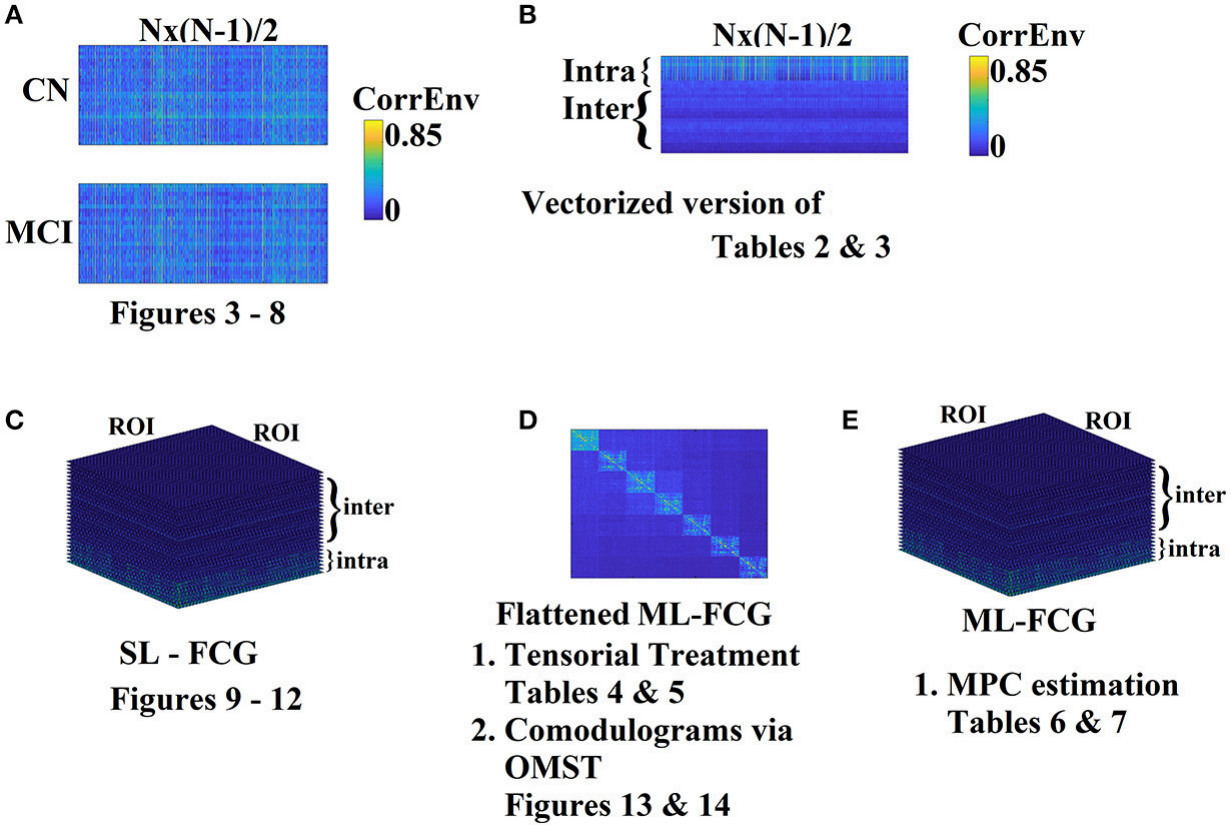
Different Representation and analytic schemes of the multiplex functional connectivity graph (FCG). **(A)** Edge-weight Feature selection approach of the SL-FCG by first vectorizing each FCG to Nx(N-1)/2 list of functional connections where N denotes the number of ROIs (here *N* = 90). **(B)** Edge-weight Feature selection approach of the ML-FCG by first vectorizing each SL-FCG to Nx(N-1)/2 list of functional connections where N denotes the number of ROIs (here *N* = 90). The dimensions of the vectorized version of the ML-FCG is intra-inter coupling modes × Nx(N-1)/2. **(C)** Tensorial treatment of each SL-FCG in both intra and inter-coupling modes. **(D)** Tensorial treatment of the flattened version of ML-FCG where in the main diagonal are tabulated the intra-frequency FCGs and in the off-diagonal the inter-frequency FCGs. The flattened version of ML-FCG has been used for the estimation of comodulograms by filtering with our OMST data-driven topological filtering method. **(E)** Estimation of MPC from the ML-FCG. SL-FCG, single layer-functional connectivity graph; ML-FCG, multi-layer-functional connectivity graph; MPC, multi-layer participation coefficient.

Most brain connectivity studies attempt to classify single-layer frequency-dependent FCG between two conditions or two groups by vectorizing the upper triangular (for undirected connectivity estimators) feature space and treat it as a high-dimensional space (Pollonini et al., 2010; Shen et al., 2010; Richiardi et al., 2011). The main drawback of the vectorized version of a FCG is that destroys the tabular representation of functional interactions among every pair of brain areas. FCG can be seen as a second-order tensor. To overcome the aforementioned limitations, we treated FCGs as *tensors* adapting tensor subspace analysis (TSA) (He et al., 2005) as a representative feature extraction algorithm. Another popular tensorial treatment of images –FCGs in computer vision area is the multi-linear PCA (ML-PCA; Lu et al., 2008). In our formulation, the tensor has dimensions of (subjects × ROIs × ROIs) as in previous works (Dimitriadis et al., 2013, 2015b,c, 2016b,c; Antonakakis et al., 2016, 2017a). TSA analysis was performed independently for ROI representation (PCA/CENT), connectivity estimator (iPLV, CorrEnv) and intrinsic coupling mode (intra/inter). Figure 2 illustrates the different representation and analytic schemes of the multiplex functional connectivity graph adapted in the present study.

#### Topological filtering of SL-FCG with OMST

Recently, we published a data-driven topological filtering approach for brain networks with the scope to reveal the true network topology from a FCG (Dimitriadis et al., 2017c). Our algorithm samples the functional connections of a FCG by iterative rounds of minimal spanning trees (MSTS; Tewarie et al., 2014) orthogonal to each other (orthogonal minimal spanning trees - OMST) and attempts to maximize the formula of global efficiency (GE) vs. the cost of the surviving selected functional connections by the OMST (Equation 1). At the 1st round the original MST is extracted; at the 2nd round the 2nd MST is estimated, which is orthogonal to the 1st. GE, and the cost of the filtered versions of the FCG is estimated by aggregating the OMST at every round. First, both measures are estimated based on the 1st MST and after that we add the OMST to the OMST of the previous round and both GE and the cost are re-estimated. The curve of GE-Cost vs. Cost is always positive and gets a maximum peak value which is the selected number of OMST rounds.

Equation (3) defines the J function that is maximized in our OMST topological filtering algorithm

(7) $$\begin{array}{r} \text{J}\left(\text{OMST}\right)=\text{GE}-\text{Cost} \end{array}$$

We have demonstrated the effectiveness of the OMST algorithm in large databases of EEG/fMRI recordings (Dimitriadis and Salis, 2017; Dimitriadis et al., 2017c), in a multi-group MEG connectivity analysis (Dimitriadis et al., 2017a) and in diffusion-based structural brain networks (Dimitriadis et al., 2017b). We topologically filtered each SL-FCG with OMST independently for ROI representation and connectivity estimator. We called hereafter the OMST version of each SL-FCG as SL-FCG^OMST^.

#### Classification scheme

For the tensorial treatment of FCG, we used SVM with RBF kernel as classifier, and the same two cross-validation schemes as above, LOOCV and 5-fold. Feature selection strategy was followed at every fold on the training set in both CV schemes. Finally, sensitivity, specificity and classification performance will be reported in both validation schemes and FCG treatment.

### Topological filtering of ML-FCG and network analysis

#### Topological filtering of ML-FCG based on omst

Prior to network analysis over ML-FCG, we topologically filtered each ML-FCG with OMST independently for each combination of ROI representation and connectivity estimator. We called hereafter the OMST version of each ML-FCG as ML-FCG^OMST^.

#### Network analysis on ML-FCG

After topological filtering, the ML-FCG based on OMST, we can extract important network metrics. These network metrics can be the global GE and the cost function of Equation (7), which assesses how efficiently the different layers (intrinsic coupling modes) are communicated in every subject. Here, we constructed the ML-FCG using the 28 single-layer FCG from the 28 different coupling modes. We didn't take into consideration any functional inter-layer relationship. Additionally, nodal GE can be estimated directly on the filtered version of ML-FCG leading to ROIs = 630 values per subject that can enter in a classification scheme as with the edge weights (see previous sections). Here, we estimated the multi-layer version of participation coefficient (MPC), which quantifies the importance of every ROI across the different layers. We adapted the multi-participation coefficient *MPC*_*i*_ in order to estimate the importance of every ROI across the ML-FCG (Battiston et al., 2014). Brain ROIs with high *MPC*_*i*_ are characteristic central hubs of the ML-FCG. The global MPC is given by the average of the *MPC*_*i*_ values:

(8) $$\begin{array}{r} MPC=\frac{1}{n}\overset{N}{\underset{i=1}{\sum }}MPC_{i}=\frac{1}{n}\overset{N}{\underset{i=1}{\sum }}\frac{M}{M-1}\left[1-\underset{\lambda }{\sum }{\left(NL{P_{i}}^{\left[\lambda \right]}\right)}^{2}\right] \end{array}$$

where stands $\left(NL{P_{i}}^{\left[\lambda \right]}\right)={k_{i}}^{\left[\lambda \right]}/o_{i}$ for *node-degree layer proportion*, which quantifies the importance of a node in a single-layer or across layers. MPC tends to be 0 when a ROI has more connections within one layer while tends to 1 when a ROI distributes their connections across the layers. Here, we used the OMST filtered versions of the 28 layers (21 intra and 7 inter-frequency FCG).

#### Comodulograms derived from the filtered ML-FCG

The topological filtering of ML-FCG with OMST algorithm (ML-FCG^OMST^) selects a specific number of connections that maximize Equation (7). These connections belong to specific layers of the ML-FCG that could be either intra or inter-frequency FCG. By counting the number of selected functional connections at every layer and dividing by their total number, we can estimated the so-called comodulograms. These comodulograms tabulate the percentage (probability) of distribution of the OMST-based connections across the different layers (7 for intra and 21 for inter-frequency coupling modes). We estimated the derived comodulograms as group-averaged for both ROI representations and connectivity estimators.

### MATLAB code and reproducibility of the results

The MATLAB code (MATHWORKS, R2017a), the raw time series and the .mat files with the static functional networks can be downloaded by the figshare site. We uploaded all the datasets under the project with the following name:

“CONNECTOMIC_BIOMARKER_MCI_MEG” project in the following links:

1. Scripts: https://figshare.com/articles/MATLAB_CODE/6127298
  doi: 10.6084/m9.figshare.6127298
2. Dataset part I (Controls):https://figshare.com/s/71a5fb9043235740a6a7
  doi: 10.6084/m9.figshare.6210158
3. Dataset part II (MCI):https://figshare.com/s/9660b976e4138853d845
  doi: 10.6084/m9.figshare.5858436
4. Pre-computed Intra and Inter-Frequency Functional Brain Networks:

- Healthy Controls:https://figshare.com/articles/Pre-computed_Functional_Brain_Networks_for_Healthy_Controls/6126866
  doi: 10.6084/m9.figshare.6126866
- MCI:https://figshare.com/articles/Pre-computed_Functional_Brain_Networks_for_MCI/6127088
  doi: 10.6084/m9.figshare.6127088

There is a memo file in the subfolder

…\code\from_raw_to_sources\data\from_sources_to_fcgs \code

called “*memo_how_to_run_the_code.m*” where one can follow the instructions step by step tp reproduce Figures 3–**14** and Tables 2–**7** and also the Supplementary Material based on PLV connectivity estimator. Running the first lines of code, one can regenerate the source time series or can jump up to the next part of the code using the pre-computed functional brain networks. Further instructions are given in the “*memo_how_to_run_the_code.m*”

**Figure 3:**
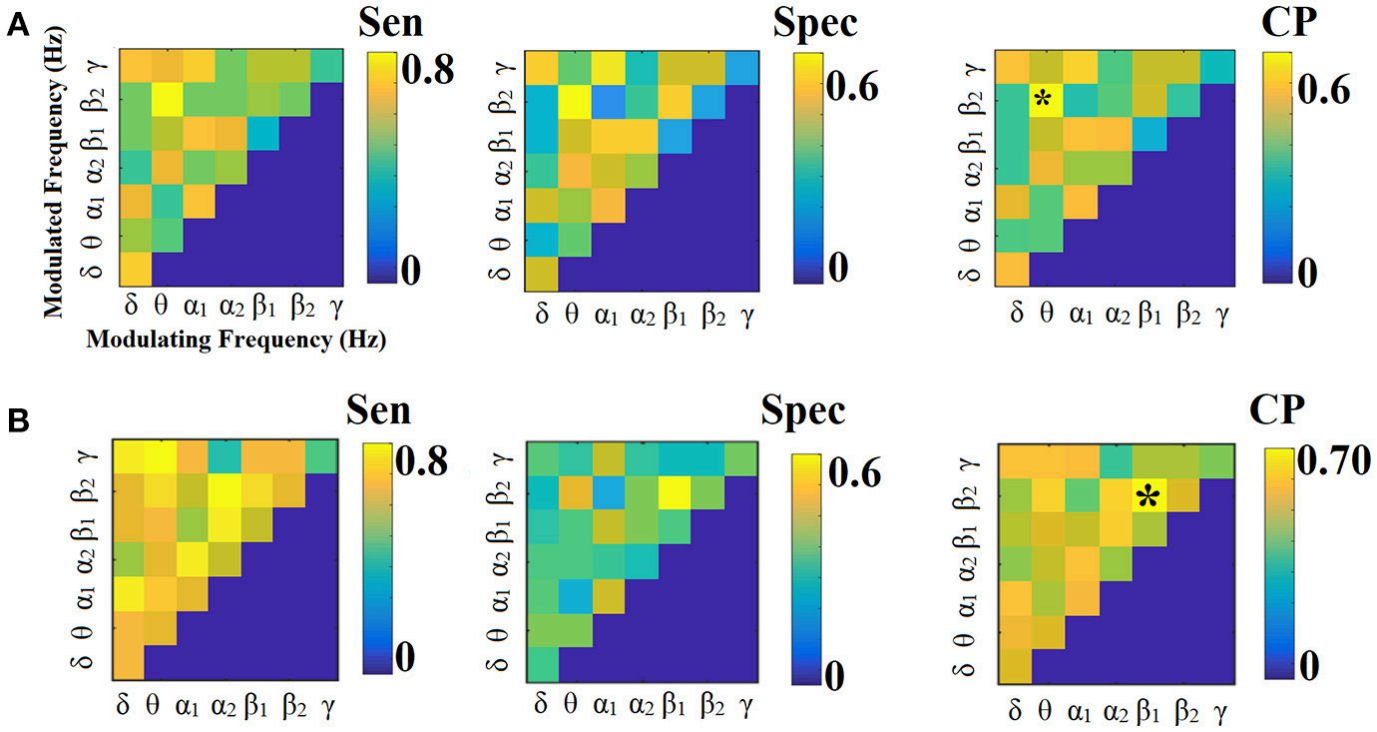
Sensitivity, specificity and classification performance of CorrEnv using PCA ROI representation and edge-weights approach of each SL-FCG. **(A)** Sensitivity, specificity and classification performance for the LOOCV. **(B)** Sensitivity, specificity and classification performance for the 5-fold CV. * denotes the best CP for each CV scheme. Sen, sensitivity; Spec, specificity; CP, Classification performance.

**Table 2:** Sensitivity, Specificity, and Classification Performance of edge-weights in ML-FCG^CorrEnv^ using the two different ROI representations (PCA and CENTroid) and two cross-validation schemes (Leave-one out cross validation and 5-fold).

		**Sensitivity**	**Specificity**	**Classification accuracy**
PCA	LOOCV	0.43	0.37	0.40
	5-FOLD	0.63 ± 0.16	0.33 ± 0.15	0.50 ± 0.09
CENT	LOOCV	0.60	0.29	0.46
	5-FOLD	0.70 ± 0.26	0.51 ± 0.29	0.60 ± 0.16

## Results

### Classification performance based on edge–weights in SL and ML FCG

#### Classification performance based on SL-FCG^CorrEnv^

**Figure 4:**
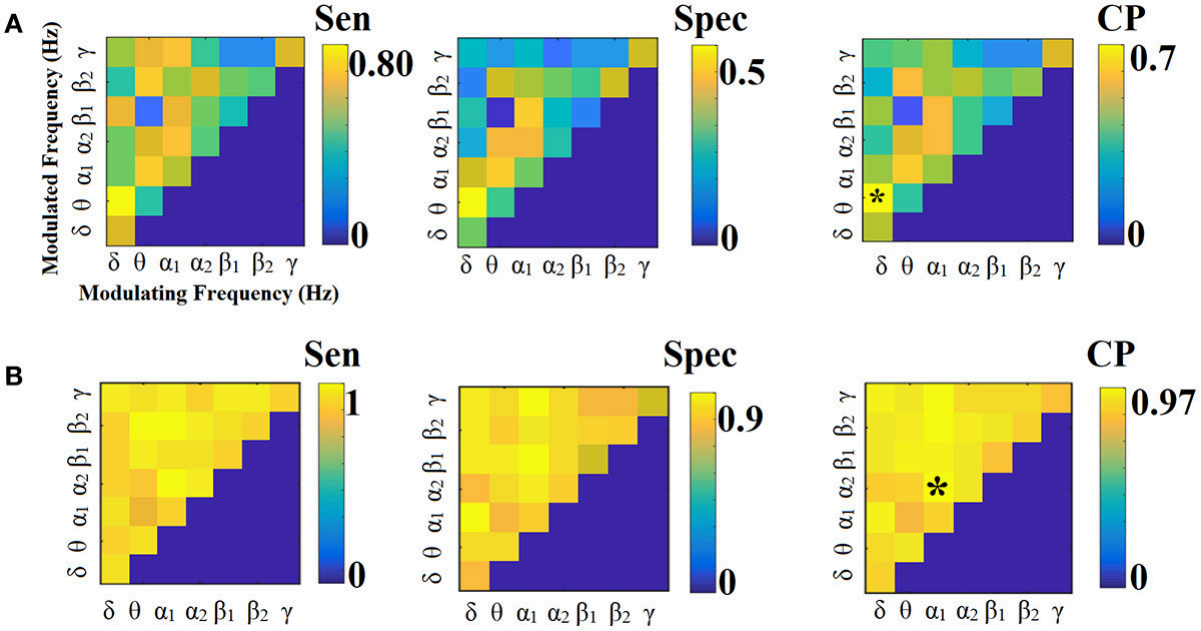
Sensitivity, specificity, and classification performance of iPLV using centroid ROI representation and edge-weights approach of each SL-FCG. **(A)** Sensitivity, specificity and classification performance for the LOOCV. **(B)** Sensitivity, specificity and classification performance for the 5-fold CV. * denotes the best CP for each CV scheme. Sen, sensitivity; Spec, specificity; CP, classification performance.

Figures 3, 4 illustrate the sensitivity, specificity and classification performance of CorrEnv using PCA and centroid ROI representation, correspondingly. The best performance for PCA representation was succeeded in θ:β_2_ for LOOCV (64%) and in β_1_:β_2_ for the 5-fold CV (72%). For the centroid representation, the best performance for LOOCV was succeeded in δ:θ (70%) and in α_1_:α_2_ for the 5-fold CV (98%). Obviously, the ROI representation alters the classification performance favoring the combination of centroid representation for CorrEnv estimator. Additionally, the CV scheme is of paramount importance for the validation of the proposed connectomic biomarker, where higher values were obtained using 5-fold CV.

**Figure 5:**
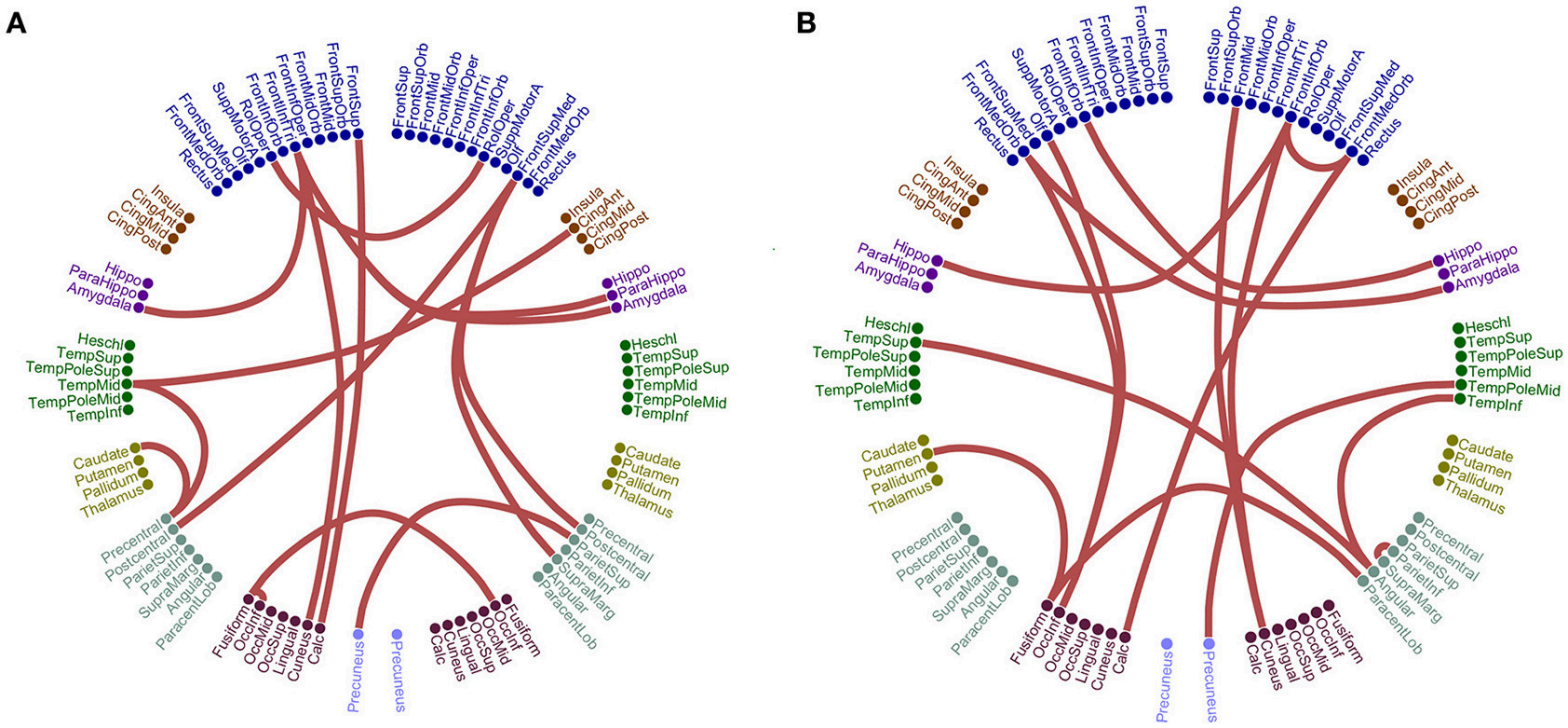
Network topology of the selected edge-weighted features using the CorrEnv connectivity estimator for β_1_:β_2_ and α_1_:α_2_. The two network topologies differ on their ROI representation approach. **(A)** PCA ROI representation for β_1_:β_2._ **(B)** Centroid ROI representation for α_1_:α_2_. The 90 ROI are illustrated circularly with 45 per hemisphere (left – right semi-circular distributions).

Figure 5 illustrates the different network topology of the selected edge-weighted features in β_1_:β_2_ / α_1_:α_2_ cross-frequency FCG based on both ROI representation schemes for the CorrEnv. Both PCA/Centroid ROI approach reveal frontal, parietal, bilateral parietal connections involving also left precuneus (Figure 5A). Centroid ROI scheme revealed bilateral temporo-parietal hemispheric connections, fronto-parietal, frontal connections involving right precuneus that improved the classification performance between the two groups (Figure 5B).

#### Classification performance based on SL-FCG^iPLV^

**Figure 6:**
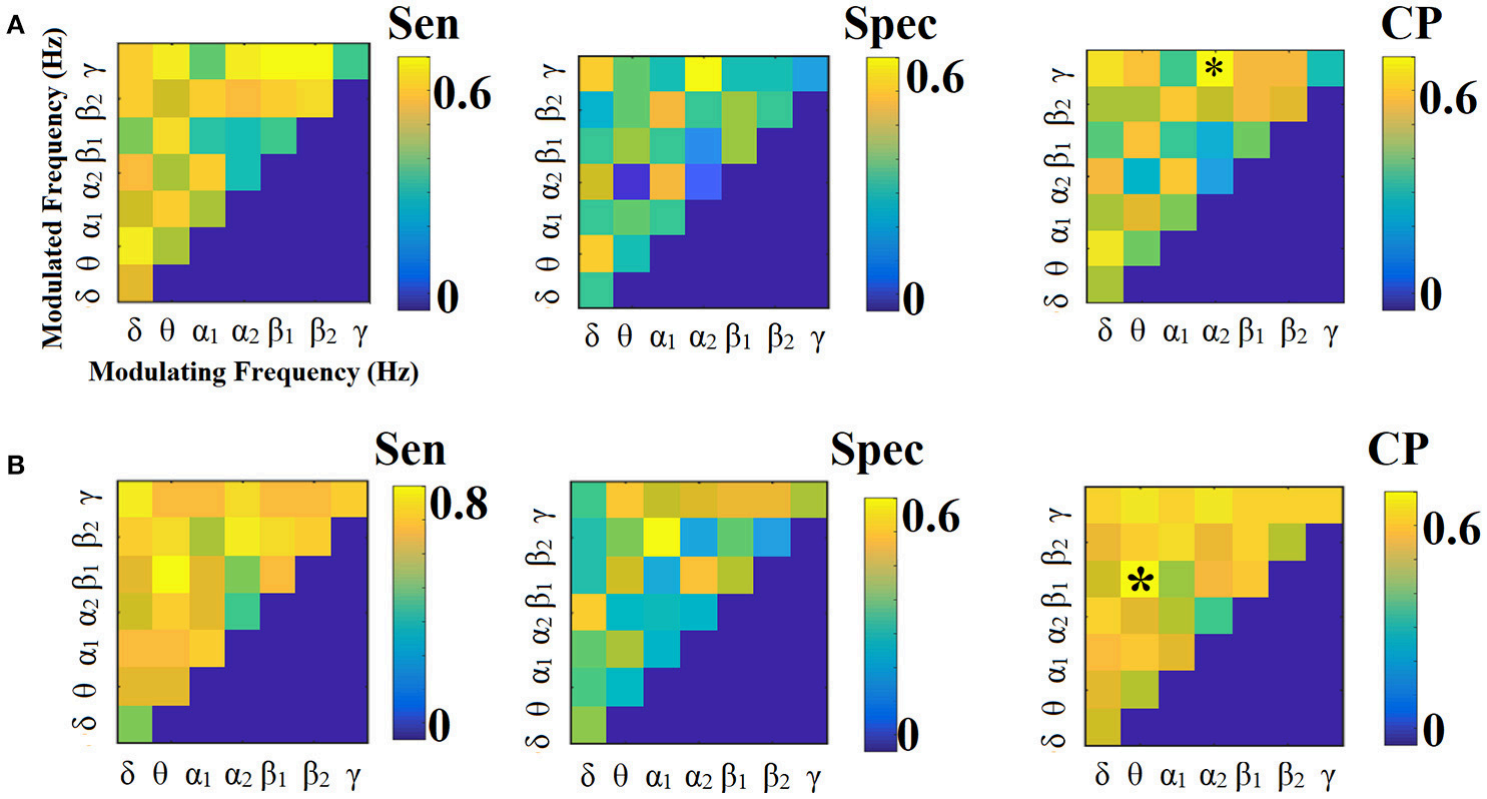
Sensitivity, specificity and classification performance of iPLV using PCA ROI representation and edge-weights approach of each SL-FCG. **(A)** Sensitivity, specificity and classification performance for the LOOCV. **(B)** Sensitivity, specificity and classification performance for the 5-fold CV. * denotes the best CP for each CV scheme. Sen, sensitivity; Spec, specificity; CP, classification performance.

**Figure 7:**
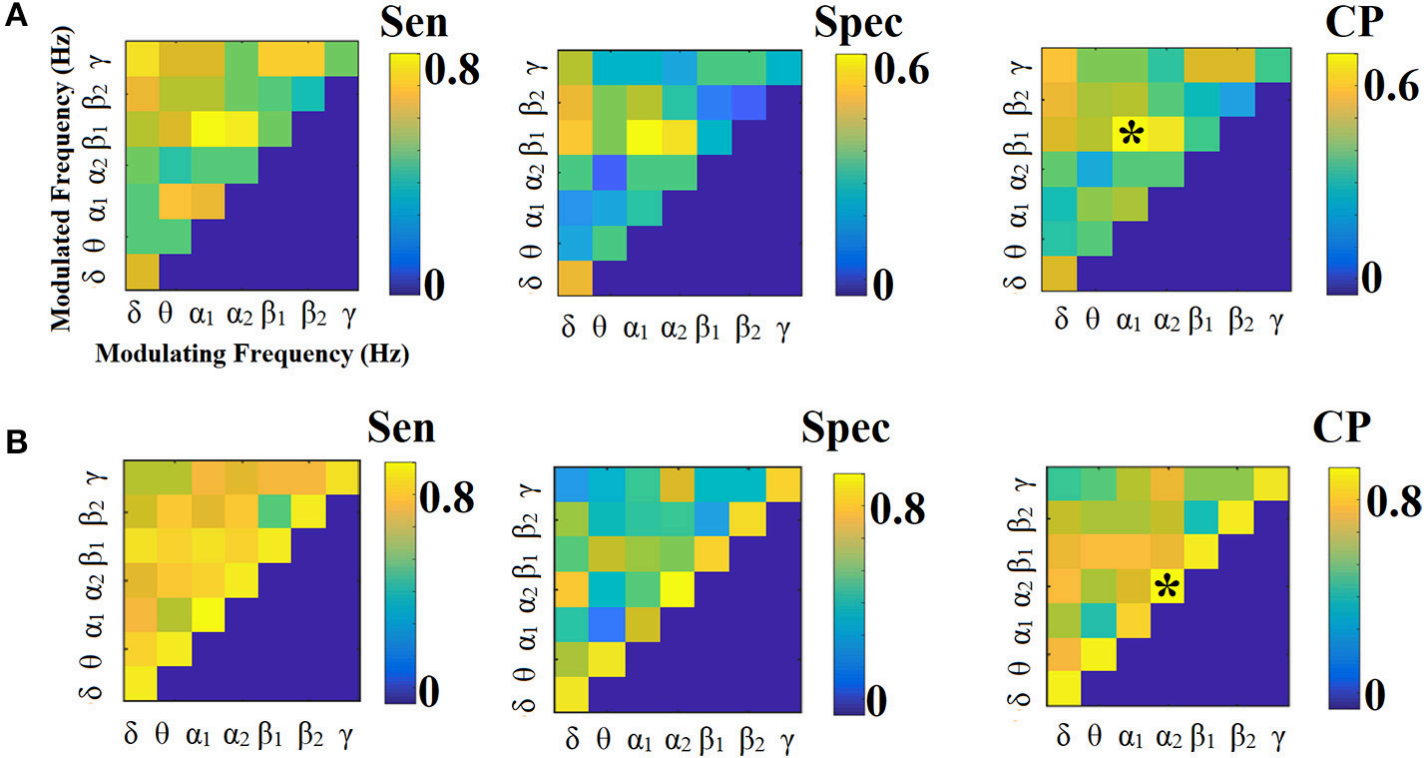
Sensitivity, specificity and classification performance of iPLV using centroid ROI representation and edge-weights approach of each SL-FCG. **(A)** Sensitivity, specificity and classification performance for the LOOCV. **(B)** Sensitivity, specificity and classification performance for the 5-fold CV. * denotes the best CP for each CV scheme. Sen, sensitivity; Spec, specificity; CP, classification performance.

Figures 6, 7 illustrate the sensitivity, specificity and classification performance of iPLV using PCA and centroid ROI representation, respectively. The best performance for PCA representation was found in α_2_:γ for LOOCV (73%) and in θ:β_1_ for the 5-fold CV (70%). For the centroid representation, the best performance for LOOCV was in α_1_:β_1_ (75%) and in α_2_ for the 5-fold CV (94%). Obviously, the ROI representation alters the classification performance favoring the combination of centroid representation for iPLV connectivity estimator. Additionally, the CV scheme is of paramount importance for the validation of the proposed connectomic biomarker, where higher values were obtained using 5-fold CV.

The classification performance of iPLV outperformed the performance of PLV favoring the use of imaginary part of PLV (see section 2 in Supplementary Material and Figures S1, S2).

**Figure 8:**
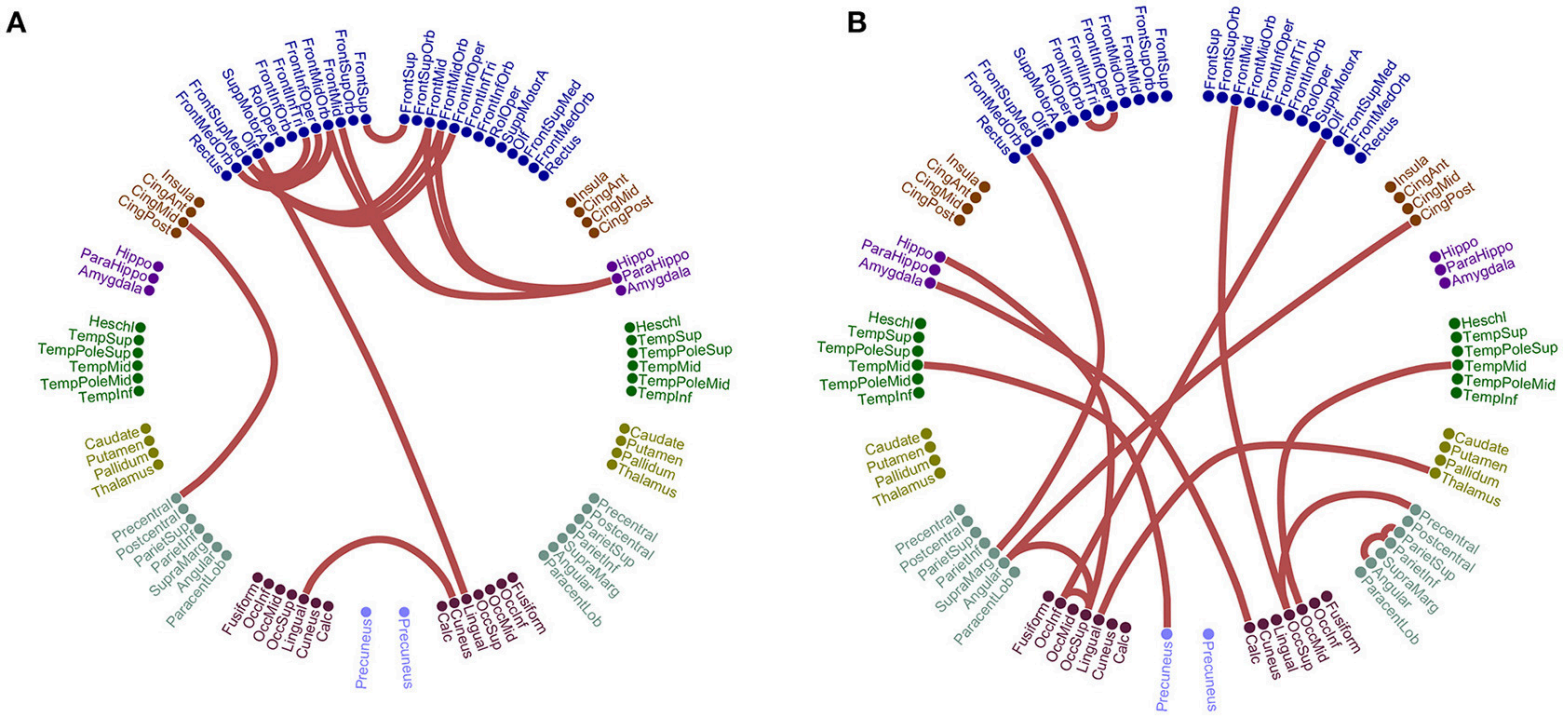
Network topology of the selected edge-weighted features using the iPLV connectivity estimator for θ:β_1_ and α_2_ intra-frequency coupling. The two network topologies differ on their ROI representation approach. **(A)** PCA ROI representation for θ:β_1_. **(B)** Centroid ROI representation for α_2._ The 90 ROI are illustrated circularly with 45 per hemisphere (left – right semi-circular distributions).

Figure 8 illustrates the different network topologies of the selected edge-weighted features in θ:β_1_ intra-frequency FCG based on PCA ROI representation schemes for the iPLV and in α_2_ for centroid ROI representation for the iPLV. Bilateral frontal connections, left fronto-temporal, bilateral-occipital, fronto-parietal and bilateral fronto-parahippo connections were revealed in PCA ROI representation (Figure 8A). Bilateral fronto-parietal, left hippo/parahippo connections with occipital brain areas, left middle temporal gyrus with precuneus and right temporo-parietal connections were revealed in centroid ROI representation (Figure 8B).

#### Classification performance based on edge –weights in ML-FCG

Following the same feature selection and cross-validation scheme in ML-FCG compared to SL-FCG, we extracted the 15 features highly consistent detected across the folds. Tables 2, 3 tabulate the sensitivity, specificity and classification performance of both connectivity estimators in both ROI representations. The classification performance was superior for the iPLV compared to CorrEnv reaching the 87% for the former compared to 55% for the latter which demonstrates the difficulty of merging the edge-weights features from SL-FCG to a ML-FCG. The classification performance of iPLV outperformed the performance of PLV favoring the use of imaginary part of PLV (see section 3 in Supplementary Material and Table S1).

**Table 3:** Same as in table 2 but for ML-FCG^iPLV^.

		**Sensitivity**	**Specificity**	**Classification Accuracy**
PCA	LOOCV	0.50	0.20	0.37
	5-FOLD	0.63 ± 0.19	0.40 ± 0.28	0.53 ± 0.11
CENT	LOOCV	0.70	0.50	0.61
	5-FOLD	0.73 ± 0.13	0.46 ± 0.08	0.61 ± 0.18

### Classification performance based on the tensorial treatment of SL-FCG

In both SL-FCG and ML-FCG formats, we extracted 6 features per dimension of the FCG which means 6 × 6 = 36 features per FCG. In both cases, the FCG were first topological filtered via the OMST filtering scheme.

#### Classification performance based on tensorial treatment of SL-FCG^CorrEnv^

**Figure 9:**
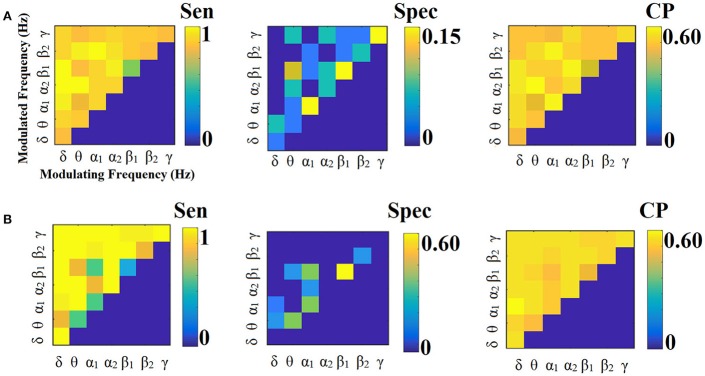
Sensitivity, specificity and classification performance of CorrEnv using PCA ROI representation and tensorial treatment of each SL-FCG. **(A)** Sensitivity, specificity and classification performance for the LOOCV and **(B)** Sensitivity, specificity and classification performance for the 5-fold CV. Sen, sensitivity; Spec, specificity; CP, classification performance.

**Figure 10:**
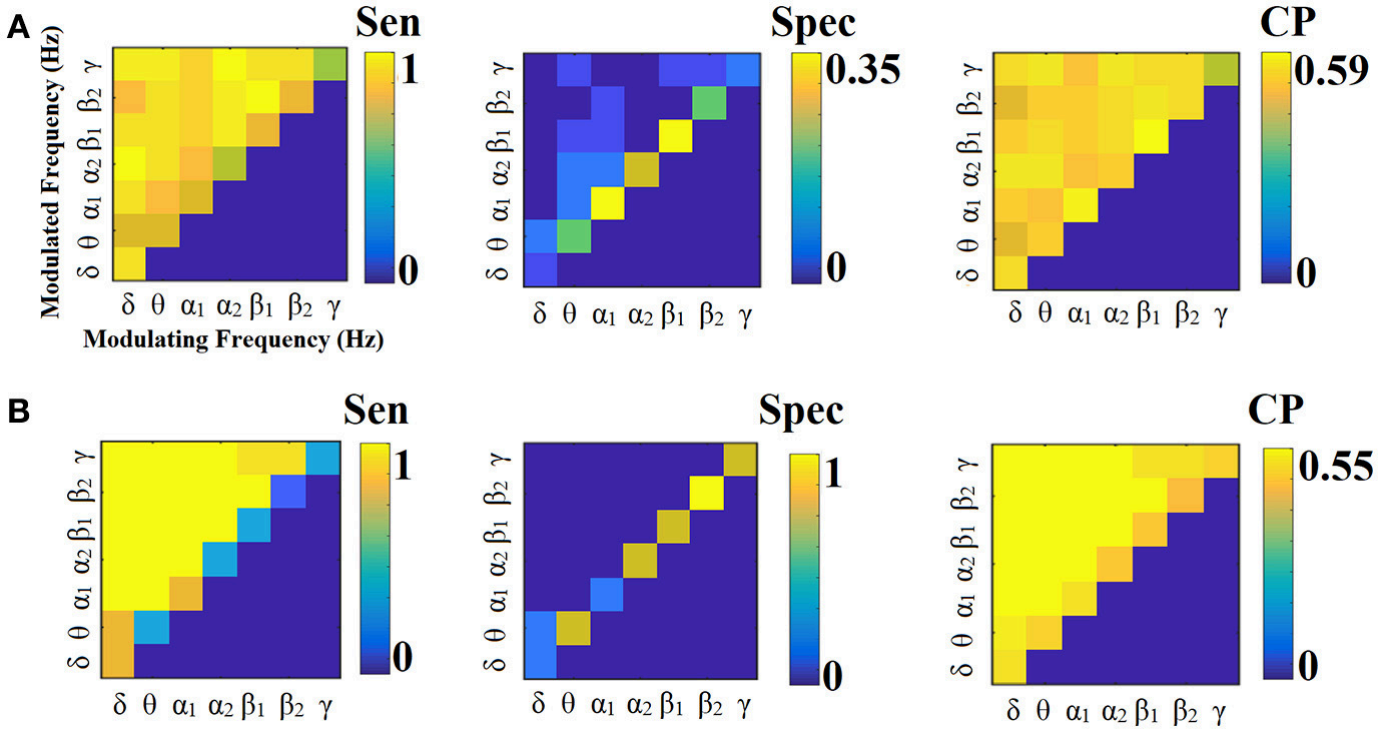
Sensitivity, specificity and classification performance of CorrEnv using Cent ROI representation and tensorial treatment of each SL-FCG. **(A)** Sensitivity, specificity and classification performance for the LOOCV. **(B)** Sensitivity, specificity and classification performance for the 5-fold CV. Sen, sensitivity; Spec, specificity; CP, classification performance.

Figures 9, 10 illustrate the sensitivity, specificity and classification performance of CorrEnv using PCA and centroid ROI representation, correspondingly. Both ROI representations and CV schemes failed to demonstrate high classification performance in every **SL-FCG**^CorrEnv^.

#### Classification performance based on tensorial treatment of SL-FCG^iPLV^

**Figure 11:**
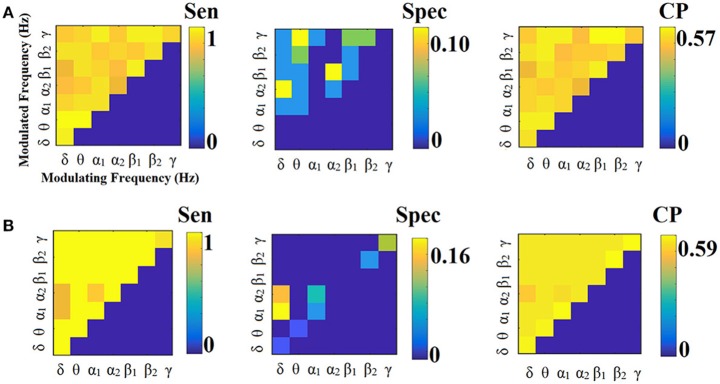
Sensitivity, specificity and classification performance of iPLV using PCA ROI representation and tensorial treatment of each SL-FCG. **(A)** Sensitivity, specificity and classification performance for the LOOCV. **(B)** Sensitivity, specificity and classification performance for the 5-fold CV. Sen, sensitivity; Spec, specificity; CP, classification performance.

**Figure 12:**
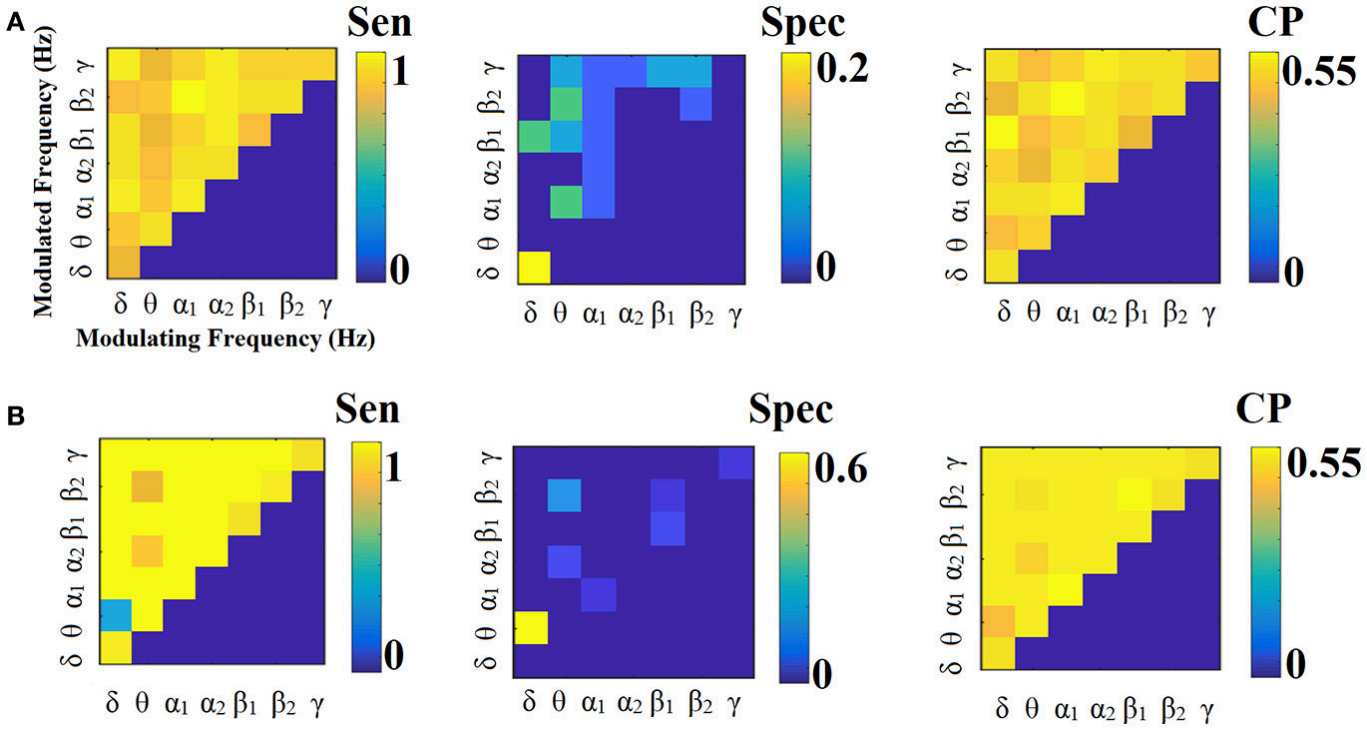
Sensitivity, specificity and classification performance of iPLV using Centroid ROI representation and tensorial treatment of each SL-FCG. **(A)** Sensitivity, specificity and classification performance for the LOOCV. **(B)** Sensitivity, specificity and classification performance for the 5-fold CV. Sen, sensitivity; Spec, specificity; CP, classification performance.

Figures 11, 12 illustrate the sensitivity, specificity and classification performance of CorrEnv using PCA and centroid ROI representation, correspondingly. Both ROI representations and CV schemes failed to demonstrate high classification performance in every **SL-FCG**^iPLV^. Classification performance based on SL-FCG^PLV^ was similar to SL-FCG^iPLV^(see Supplementary Material in section 4 and Figures S4, S5).

#### Classification performance based on the tensorial treatment of ML-FCG^OMST^

We followed the same tensorial feature extraction and cross-validation scheme in ML-FCG as the ones used for each SL-FCG. In both cases, the classification performance were on the level of by chance (50%), which demonstrates the difficulty of merging the edge-weights features from SL-FCG to a ML-FCG. In both estimators (see Tables 4, 5), the classification performance were similar compared to each SL-FCG using the tensorial treatment of the FCG but our results were too low compared to the edge-weights approach. Classification performance based on ML-FCG^PLV^ was similar to ML-FCG^iPLV^ and to ML-FCG^CorrEnv^ (see Supplementary Material in section 5 and Table S1).

**Table 4:** Sensitivity, specificity and classification performance of the tensorial treatment of ML-FCG^CorrEnv^ using two ROI representation and two cross-validation schemes.

		**Sensitivity**	**Specificity**	**Classification accuracy**
PCA	LOOCV	0.70	0.00	0.38
	5-FOLD	1.00 ± 0.00	0.00 ± 0.00	0.55 ± 0.02
CENT	LOOCV	0.86	0.04	0.50
	5-FOLD	1.00 ± 0.00	0.00 ± 0.00	0.55 ± 0.02

**Table 5:** Same as in table 4 but for ML-FCG^iPLV^.

		**Sensitivity**	**Specificity**	**Classification accuracy**
PCA	LOOCV	0.90	0.04	0.51
	5-FOLD	1.00 ± 0.00	0.09 ± 0.12	0.59 ± 0.07
CENT	LOOCV	1.00	0.00	0.55
	5-FOLD	1.00 ± 0.00	0.00 ± 0.00	0.55 ± 0.02

### Network analysis and comodulograms of ML-FCG^OMST^

#### Network analysis of the ML-FCG^OMST^

We estimated MPC on the ML-FCG^OMST^ based on the degree of each node at every single layer. Across both connectivity estimators, ROI representation and cross-validations schemes, the best performance was above by chance (Tables 6, 7). The common selected feature across ROI representation and cross-validation scheme for iPLV estimator was the left superior frontal gyrus while for CorrEnv were the left inferior parietal lobule, the left paracentral lobule and left temporal superior gyrus. Classification performance and specificity based on MPC extracted from ML-FCG^PLV^ was lower compared to both ML-FCG^iPLV^ and to ML-FCG^CorrEnv^ while sensitivity was higher (see Supplementary Material in section 6.1 and Table S1).

**Table 6:** Sensitivity, specificity and classification performance of MPL estimated over the ML-FCG^CorrEnv^ using two ROI representation and two cross-validation schemes.

		**Sensitivity**	**Specificity**	**Classification accuracy**
PCA	LOOCV	0.33	0.29	0.31
	5-FOLD	0.63 ± 0.19	0.38 ± 0.16	0.52 ± 0.15
CENT	LOOCV	0.60	0.66	0.62
	5-FOLD	0.63 ± 0.19	0.38 ± 0.16	0.52 ± 0.15

**Table 7:** Same as in table 6 but for ML-FCG^iPLV^.

		**Sensitivity**	**Specificity**	**Classification accuracy**
PCA	LOOCV	0.76	0.41	0.61
	5-FOLD	0.66 ± 0.21	0.37 ± 0.28	0.53 ± 0.18
CENT	LOOCV	0.66	0.16	0.44
	5-FOLD	0.66 ± 0.21	0.37 ± 0.28	0.53 ± 0.18

#### Comodulograms of the ML-FCG^OMST^

**Figure 13:**
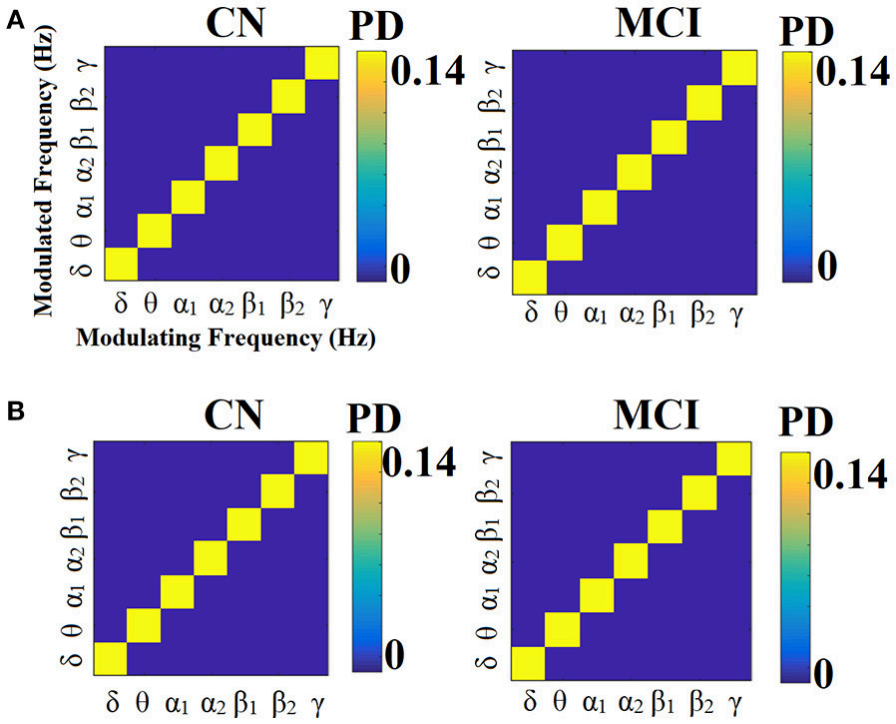
Group-averaged comodulograms derived from ML-FCG^CorrEnv^. **(A)** PCA ROI representation. **(B)** Centroid ROI representation. PD, probability distribution.

**Figure 14:**
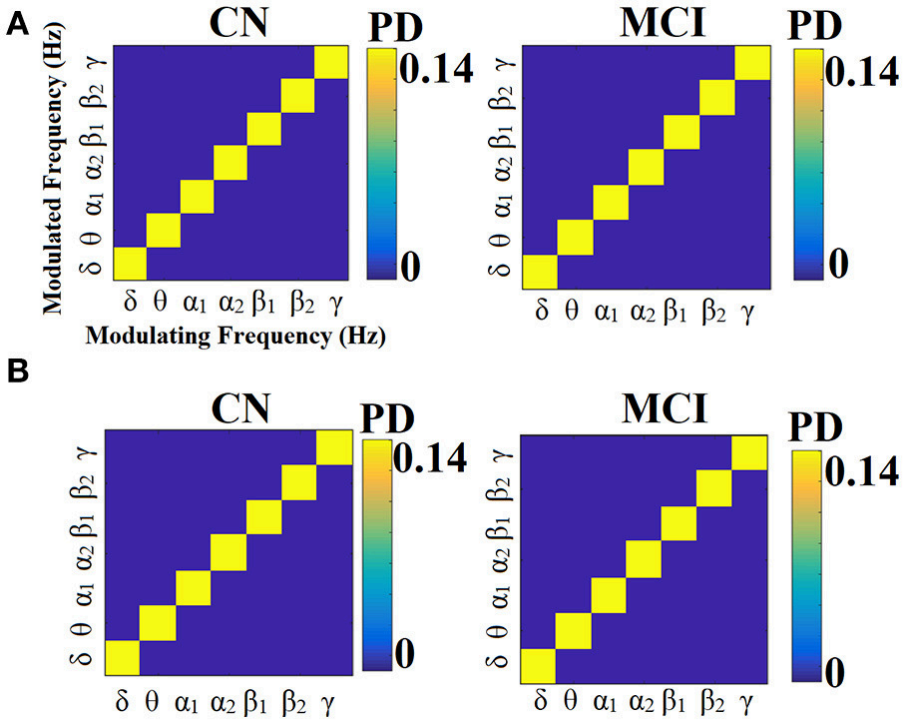
Group-averaged comodulograms derived from ML-FCG^iPLV^. **(A)** PCA ROI representation. **(B)** Centroid ROI representation. PD, probability distribution.

Figures 13, 14 illustrate the group-averaged comodulograms for CorrEnv and iPLV correspondingly. Each 2D plots demonstrate the probability distribution of selected edges via the OMST filtering approach across the multi-layer. The in-diagonal cells in comodulograms keep the PD of the functional connections within each layer (intra-frequency coupling) while the off-diagonal cells keep the PD of the functional connections between the layers (cross-frequency couplings). Even though it is not clear from the color-coded, there are on average 8 connections between every pair of δ modulator with the rest of modulated frequencies in every case (ROI representations × connectivity estimators). It is obvious in all cases (ROI representation × connectivity estimators) that the basic modulating frequency is the δ brain rhythm (Figures 13A, 14). δ is the modulating frequency that serves as central hub that connects the multi coupling modes layers of the ML-FCG. PD ROI representation didn't affect the contribution of intra/inter frequency-coupling modes in both CorrEnv and iPLV connectivity estimators.

## Discussion

Here, we demonstrated a framework to build a highly efficient connectomic biomarker for a brain disease (here, MCI). The whole research is novel and unique, attempting to reveal the difficulties and the pitfalls of analyzing neuroimaging recordings with main scope to build a connectomic biomarker.

The whole analysis focused on a static functional connectivity analysis at the source level after beamforming MEG resting-state activity in healthy controls and MCI subjects. We adopted the well-known AAL template with 90 ROIs that represent the nodes of the FCG. Two different preprocessing choices in ROI representation were used, the PCA and the centroid approach. For functional connectivity estimators, we employed CorrEnv and iPLV. Both estimators were adopted for the construction of intra and inter-frequency coupling modes FCG. Going one step further, the different versions of FCG were analyzed as a SL-FCG and as a ML-FCG. For the construction of a high efficient connectomic biomarker, we followed two different scenarios in both SL-FCG and ML-FCG. Functional connections in the tabulated FCG were further analyzed as single edge-weighted features and the whole FCG as a 2D tensor. In the former case, the original FCG was treated in the fully-weighted versions while in the latter case, we first filter both SL-FCG and ML-FCG via OMST data-driven topological filtering approach (Dimitriadis et al., 2015a, 2017a,b; Dimitriadis and Salis, 2017). Finally, we applied a network analysis on the filtered version of ML-FCG^OMST^ to reveal the patterns of dominant intrinsic coupling modes of each group and the efficiency of the communication across the multi-layers.

The results of the present study can be summarized as follow, based on the classification performance of the 5-fold CV scheme:

1. Edge-weighed feature selection strategy outperformed the tensorial treatment of SL-FCG and ML-FCG
2. Based on CorrEnv, the highest CP (98%) was obtained using centroid ROI representation in α_1_:α_2_ FCG
3. Based on iPLV, the highest CP (94%) was obtained using centroid ROI representation in α_2_ FCG
4. ROI representation affects the topology of the selected edge-weights features in both connectivity estimators (Figures 5, **8**)
5. Centroid ROI representation outperforms PCA in both connectivity estimators
6. Edge-weighted feature selection in ML-FCG favors the iPLV estimator over CorrEnv but the CP were too low.
7. Classification performance based on MPC with both connectivity estimators are slightly above by chance (52%)
8. Imaginary part of PLV outperformed PLV in every experiment performed in the current study supporting further its use as a valuable connectivity estimator

The network topology of the edge-weighted feature selection approach revealed different patterns according to the ROI representation and the connectivity estimator. Regarding CorrEnv, the best performance for PCA representation was succeeded in θ:β_2_ for LOOCV (64%) and in β_1_:β_2_ for the 5-fold CV (72%) (Figure 3). For the centroid representation, the best performance for LOOCV was succeeded in δ:θ (70%) and in α_1_:α_2_ for the 5-fold CV (98%) (Figure 4).

Figure 5 illustrates the different network topology of the selected edge-weighted features in β_1_:β_2_ / α_1_:α_2_ cross-frequency FCG based on both ROI representation schemes for the CorrEnv. Both PCA/Centroid ROI approach reveal frontal, parietal, bilateral parietal connections involving also left precuneus (Figure 5A). Centroid ROI scheme revealed bilateral temporo-parietal hemispheric connections, fronto-parietal, frontal connections involving right precuneus that improved the classification performance between the two groups (Figure 5B).

In contrast, the best performance for PCA representation was found in α_2_:γ for LOOCV (73%) and in θ:β_1_ for the 5-fold CV (70%) (Figure 6). For the centroid representation, the best performance for LOOCV was in α_1_:β_1_ (75%) and in α_2_ for the 5-fold CV (94%) (Figure 7). Obviously, the ROI representation alters the classification performance favoring the combination of centroid representation for iPLV connectivity estimator. The classification performance of iPLV outperformed the performance of PLV favoring the use of imaginary part of PLV (see section 2 in Supplementary Material and Figures S1, S2).

Figure 8 illustrates the different network topologies of the selected edge-weighted features in θ:β_1_ intra-frequency FCG based on PCA ROI representation schemes for the iPLV and in α_2_ for centroid ROI representation for the iPLV. Bilateral frontal connections, left fronto-temporal, bilateral-occipital, fronto-parietal and bilateral fronto-parahippo connections were revealed in PCA ROI representation (Figure 8A). Bilateral fronto-parietal, left hippo/parahippo connections with occipital brain areas, left middle temporal gyrus with precuneus and right temporo-parietal connections were revealed in centroid ROI representation (Figure 8B).

Of paramount important is the connection between left precuneus and left superior occipital pole (Figure 8B). A recent study using fMRI showed the effect of hippocampus' functional connections in episodic memory for MCI subjects (Papma et al., 2017). Both schemes revealed a bilateral parietal connection with the involvement of precuneus with post cingulum (Figure 8A) and with frontal medial orbital (Figure 8B). Another recent study using rs-fMRI recordings and seed-based FC analysis revealed the significant role of precuneus as a hub area where its pattern of connections is altered in MCI and AD subjects (Yu E. et al., 2017).

The proposed multivariate connectomic biomarker for MCI based on beamformed activity at resting-state and the edge-weighted scenario (Figures 5–8) was built with functional connections that are located between and within ROIs part of default-mode, fronto-parietal, and cingulo-opercular network. Our results further support the significant role of these three functional brain networks in both healthy and disease conditions (Cole et al., 2014; Sheffield et al., 2015).

We reported higher classification performance based on iPLV compared to PLV (Supplementary Material). Recent studies demonstrated that imaginary part of *PLV* (*iPLV*) can remove artificial interactions bu it cannot eliminate spurious interactions if the true coupling has non-zero phase lag (Palva et al., 2018; Wang et al., 2018). They finally suggest that hyperedge bundling can significantly decreases graph noise by minimizing the false-positive to true-positive ratio (Wang et al., 2018).

A recent study using resting state MEG recordings in controls and AD patients reported the diagnostic power of MPC derived from multi-layer FCG. The multi-layer graph consisted only on intra-frequency coupling modes, while the different layers were artificially linked with connections between homolog rain ROIs. They gave an increased classification accuracy of 74% and a sensitivity of 80% based on iPLV (Guillon et al., 2017). Here using 28 layers of intra and inter-frequency coupling FCG, the best performance for the MPC was obtained using the CorrEnv with both ROI representation reaching the 64% with 83% of sensitivity.

Recently, we introduced the notion of integrated FCG (I-FCG) where at every pair of nodes, we assigned a dominant coupling mode across both intra and inter-frequency couplings. The whole procedure has demonstrated its effectiveness in both static and dynamic M/EEG networks in healthy controls, dyslexia and mild traumatic brain injury (Dimitriadis et al., 2015b, 2016b; Antonakakis et al., 2016, 2017a; Dimitriadis, 2016a; Dimitriadis and Salis, 2017). The whole approach used surrogate analysis and Bonferroni correction in order to uncover the dominant coupling mode per pair of ROI. This I-FCG can be seen as a single-layer version of the ML-FCG where we keep both the weights and the preferred coupling mode. Due to limitations of running the scripts by the reviewers for evaluation, we excluded it for demonstration but we are in preparation of new manuscripts based on the same cohort in order to include I-FCG and surrogate analysis to the whole pipeline.

We estimated for both intra and inter-frequency coupling two well-known estimators: the CorrEnv and iPLV. In the special case of CFC, we estimated the popular PAC using iPLV where the phase of the low frequency rhythm modulates the amplitude of the higher frequency oscillation (Canolty and Knight, 2010; Dimitriadis et al., 2015a, 2016a,d; Antonakakis et al., 2016, 2017a,b; Dimitriadis and Salis, 2017). Human spontaneous activity is shaped by the CFC that coordinates the activity between distant and local brain areas that function on their preferred oscillations (Florin and Baillet, 2015). PAC has been reported in many conditions and for many cross-frequency pairs like in δ:δ (Lakatos et al., 2005), δ:α (Ito et al., 2013), δ:β (Nakatani et al., 2014), δ:γ (Szczepanski et al., 2014), θ:α (Cohen et al., 2009), θ:β (Cohen et al., 2009; Nakatani et al., 2014), θ:γ (Dürschmid et al., 2013; Florin and Baillet, 2015), α:β (Sotero et al., 2015), α:γ (Spaak et al., 2012), and β:γ (de Hemptinne et al., 2013). Although in many experimental studies, authors focused on only one cross-frequency pair, the majority of them can be detected simultaneously in a single condition (Sotero et al., 2015).

By integrated both intra and the various inter-frequency coupling modes into a static and dynamic FCG is of paramount importance. In our previous studies, we demonstrated also how comodulograms of the dominant intrinsic coupling modes can discriminate healthy controls from disease groups in both static and dynamic FCG (Dimitriadis et al., 2010, 2015a, 2016a,d; Antonakakis et al., 2016, 2017a,b; Dimitriadis and Salis, 2017). However, it is significant to analyse intra and PAC interactions via multivariate approach in order to reveal the indirect interactions and the direction of the information transmission between the brain areas. We have already started to work on this approach and we will report our findings on the same open dataset using multivariate information theoretic tools (Lizier et al., 2011).

Multiplexity of human brain dynamics is a recent hot topic in neuroscience. Recent advances in both structural and functional neuroimaging integrated neuroscience, informatics, mathematics and physics into a single goal, how the brain functions in healthy states and how dysfunctions in various diseases. Here, we accessed the multiplexity of human brain via static functional brain networks across various coupling modes. We built multi-layer FCG employing both intra and cross-frequency coupling FCG with main scope to estimate the complexity of human brain activity across spatial and functional scales. We estimated the MPC as a network metric that quantifies the importance of every ROI across the multi-layers. The estimation of MPC based on ML-FCG with no inter-layer connections (Tables 6, 7; Guillon et al., 2017; Yu M. et al., 2017). Complementary, a flattened ML-FCG version has been constructed with connections between the intra-frequency layers the so-called cross-frequency coupling estimates. Using OMST filtering scheme, we selected the significant trend of dominant coupling modes across both spatial and frequency scales illustrated in the comodulograms (Figures 13, 14). Both techniques are important to be added in the alternative network analysis tools for estimating the multiplexity of human brain dynamics.

The aforementioned statement is applicable in analyzing the intra and inter-frequency interactions between the amplitudes of the source time series. Multivariate information theoretic connectivity tools will be applied from our team complementary to the phase interactions. Our attempt was to demonstrate the difference, the commonalities and the complementarity of the basic connectivity estimators in both amplitude and phase domain.

A recent study concluded that the network topology, the CFC and the intra-frequency interactions shaped the PAC generation in a cortical column using a novel neural mass model (Sotero, 2016). Here, in order to reduce the computational time needed to run the pipeline from the reviewers in order to evaluate the whole analysis, we did not run surrogate analysis. Surrogate analysis is important to statistical filter out the spurious connections (Aru et al., 2015) and to reveal the true connections prior to the topological filtering OMST scheme.

Finally, we would like to state that a connectomic biomarker could be build by integrating SL or ML-FCG from different connectivity estimators especially if they estimate functional connectivity in amplitude and phase domains.

### Limitations of the current analysis

One of the basic limitations of this study is the lack of surrogate analysis. We have already reported that surrogate analysis tailored to each connectivity estimator and interactions (intra and inter) should be reported in every brain connectivity study. In the case of searching the best features—functional weights that increase the classification performance between two groups, we assumed that all the connection exist in every single subject. This is not true, yet there are many studies that report their results under this assumption. Surrogate analysis can be seen as a statistical filtering (pruning) of the whole network, whereby only the significant links at a certain threshold are preserved. After first applying the statistical filtering (surrogates) and topological filtering (e.g., OMST), the true network topology can emerge from each of the subject-specific FCG. This practically means that only a small amount of connections co-exist across our dataset. In that case, two options can be used to design a connectomic biomarker. The first one is to handle the FCG as a tensor, as we demonstrated here, and to estimate nodal network metrics such as global/local efficiency. In the second case, our features will be the nodal network metrics instead of the single-edge weights.

In previous studies, we applied the tensorial extraction algorithm on the original MEG sensor space and we reported significant results. However, here the tensorial treatment of the FCG in both the single and multi-layer options did not work properly. This misclassification of the tensors could be attributed to many pitfalls. Here, we used a fixed anatomical template for every subject in both groups, which is common in functional neuroimaging while the number of ROIs maybe too low to support the computational power of the FCG-based approach. Another interpretation could be the missing of surrogate analysis and the use of bivariate connectivity estimators.

It is important to stress the need to evaluate the proposed algorithmic scheme in a second blind dataset and in a follow—up cohort with MCIs that are either stable or progressed to AD (López et al., 2014b, 2016). Additionally, a reliable connectomic biomarker should be tested across multi-site recordings (Maestú et al., 2015), most desirably including different MEG systems (CTF - ELEKTA).

## Conclusions

We demonstrated how different preprocessing steps in the definition of the representative time series of each ROI, the selection of a connectivity estimator and the formulation of the FC graph could alter the outcome of the design of a connectomic biomarker. We demonstrated two different approaches to study the functional brain network, as a vector of single functional weights or as a unit – 2D matrix, where more tools should be added to our list such as tensorial extraction algorithms. Additionally, it is always important, whenever possible, to evaluate the proposed connectomic biomarkers in a second blind dataset, in order to increase the generalization of the proposed algorithm and to test it across multi-site cohorts with the same or different MEG system. Only under this umbrella of effort, a reliable clinically-usable connectomic biomarker can be proposed in the neuroscience community.

We strongly encouraged the neuroscience MEG community to add on their analysis different ROI representation, connectivity estimators and also both intra and cross-frequency coupling mechanisms should be included. The take home message from this seminar work is that centroid outperformed PCA independently of the connectivity estimator while the treatment of every edge as a unit compared to the tensorial treatment gave better results. We hypothesize that the number of ROIs using the AAL probably are not enough to give good performance for the tensorial treatment of functional brain networks and a more fine-grained parcellation scheme should be incorporated in the pipeline. Finally, we reported results from the famous MPC where two research groups revealed significant differences between healthy controls and AD group. However, the performance of MPC in our case employing also cross-frequency layers was lower than the edge-weighed approach. Finally, dynamic network connectivity analysis could reveal more discriminative profiles of both groups that can better discriminated compared to static connectivity analysis and also validated in external blind datasets across sites and MEG scanners.

## Author contributions

SD: conception of the research, methods and design, and drafting the manuscript; SD, ML, RB, PC, FM, EP data analysis; ML data acquisition; AM subject recruitments and validation of their neurological statement; ML, RB, PC, AM, FM, EP critical revision of the manuscript; every author read and approved the final version of the manuscript.

### Conflict of interest statement

The authors declare that the research was conducted in the absence of any commercial or financial relationships that could be construed as a potential conflict of interest.

## Abbreviations

MCI: Mild cognitive impairment
AD: Alzheimer's Disease
FC: Functional Connectivity
EEG: Electroencephalography
MEG: Magnetoencephalography
CFC: Cross Frequency Coupling
PAC: Phase - to - Amplitude Coupling
FCG: Functional Connectivity Graph
SL-FCG: Single-Layer Functional Connectivity Graph
ML-FCG: Multi-Layer Functional Connectivity Graph
LOOCV: Leave-One-Out Cross-Validation
CorrEnv: Correlation of the Envelope
iPLV: imaginary part of Phase Locking Value
TSA: Tensor Subspace Analysis
MPC: Multi-Layer Participation Coefficient
SOBI: Second Order Statistics
AAL: Automatic Anatomical Labeling
LCMV: Linearly Constrained Minimum Variance
PCA: Principal Component Analysis
Cen: Centroid.
