# Supplementary material for: How to Build a Functional Connectomic Biomarker for Mild Cognitive Impairment From Source Reconstructed MEG Resting-State Activity: The Combination of ROI Representation and Connectivity Estimator Matters
Source: Front Neurosci. 2018 Jun 1;12:306. doi: 10.3389/fnins.2018.00306 (PMC5992286; doi:10.3389/fnins.2018.00306)
Supplement: Supplementary file 1 [file Data_Sheet_1.DOCX]

**SUPPLEMENTARY MATERIAL**

**How to Build a Functional Connectomic Biomarker for Mild Cognitive Impairment from Source Reconstructed MEG Resting-State Activity: The Combination of ROI Representation and Connectivity Estimator Matters**

**Dimitriadis SI^1-5*^,** María Eugenia López^6,7^, Ricardo Bruña^7^, Pablo Cuesta^7,8^, Alberto Marcos^8^, Fernando Maestu^6,7^, Ernesto Pereda^7,9^

^1^Division of Psychological Medicine and Clinical Neurosciences, School of Medicine, Cardiff University, Cardiff, United Kingdom

^2^Cardiff University Brain Research Imaging Centre (CUBRIC), School of Psychology, Cardiff University, Cardiff, United Kingdom

^3^School of Psychology, Cardiff University, Cardiff, United Kingdom

^4^Neuroinformatics Group, Cardiff University Brain Research Imaging Centre, School of Psychology, Cardiff University, Cardiff, United Kingdom

^5^Neuroscience and Mental Health Research Institute, Cardiff University, Cardiff, United Kingdom

^6^ Department of Basic Psychology II, Complutense University of Madrid, Spain

^7^ Lab. of Cognitive and Computational Neuroscience, CTB, Madrid, Spain

^8^ Department of Neurology, San Carlos University Hospital, Madrid, Spain

^9^ Electrical Engineering and Bioengineering group, Department of Industrial Engineering & IUNE, Universidad de La Laguna, Tenerife, Spain

*Corresponding author : (Dr.Dimitriadis Stavros ; [stidimitriadis@gmail.com](mailto:stidimitriadis@gmail.com) ; [DimitriadisS@cardiff.ac.uk](mailto:DimitriadisS@cardiff.ac.uk) )

Institute of Psychological Medicine and Clinical Neurosciences, Cardiff University School of Medicine, Cardiff, UK

**Tel**:+44- 029 225 10258

1. **Exploring the Performance of PLV as a potential biomarker for MCI**

In this section, we presented the performance of original real value PLV on the binary classification of healthy control individuals versus MCI. The whole analysis is the same as in the main manuscript.

The original *PLV* is defined as follows:

$$PLV=\frac{1}{T}\left| \sum_{t=1}^{T} e^{i\left( \varphi F\left( t \right)-\varphi F\left( t \right) \right)}) \right| (1)$$

**2. Classification Performance based on SL-FCG^PLV^**

S.Fig. 1 and 2 illustrate the sensitivity, specificity and classification performance of PLV using PCA and centroid ROI representation, respectively. The best performance for PCA representation was found in θ:θ for LOOCV (70%) and in θ:α_2_ for the 5-fold CV (60%). For the centroid representation, the best performance for LOOCV was in α_2_:β_1_ (77%) and in α_1_:α_1_ for the 5-fold CV (90%). Obviously, the ROI representation alters the classification performance favouring the combination of centroid representation for PLV connectivity estimator. Additionally, the CV scheme is of paramount importance for the validation of the proposed connectomic biomarker, where higher values were obtained using 5-fold CV. Finally, by direct comparison of S.Fig.2 and Fig.4, iPLV outperforms PLV in sensitivity,specificity and performance across the various coupling modes favouring the notion of the imaginary part of PLV.


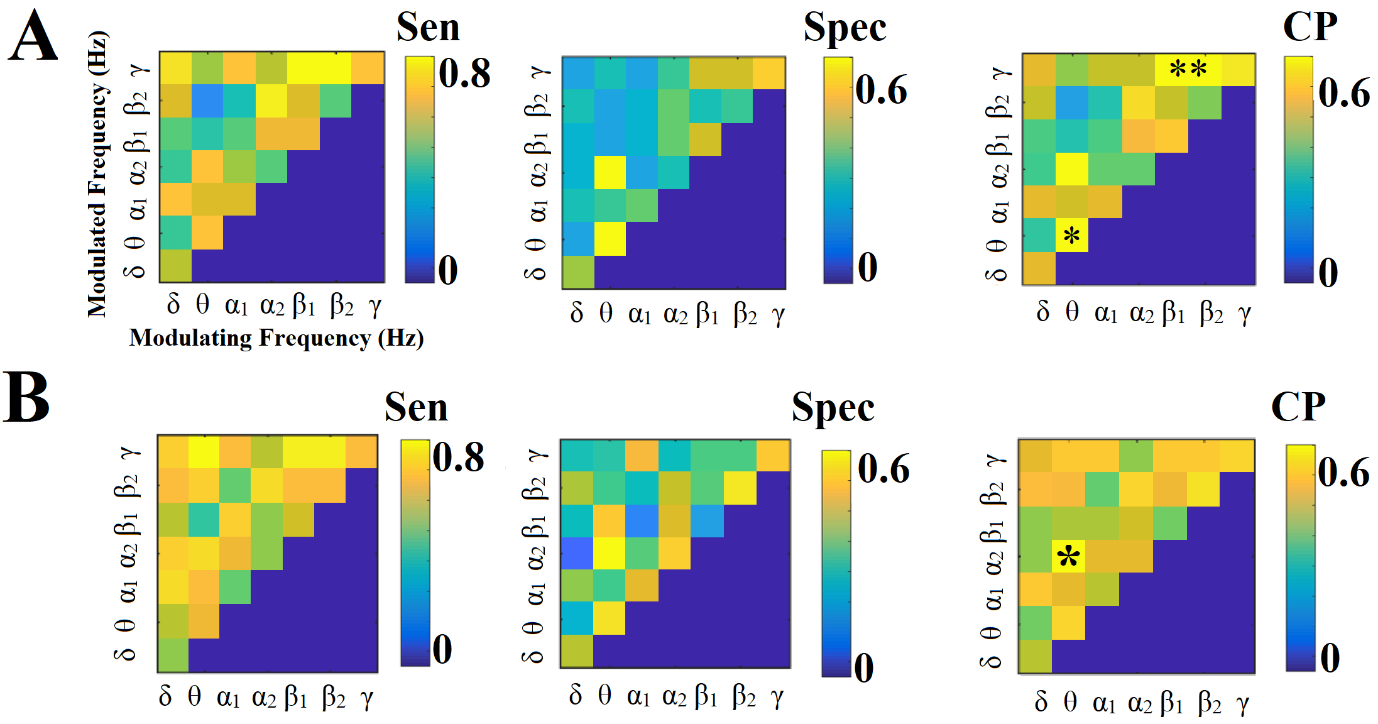


**S.Fig. 1. Sensitivity, Specificity and Classification Performance of PLV using PCA ROI representation and edge-weights approach of each SL-FCG.**

A)Sensitivity, Specificity and Classification Performance for the LOOCV and

B) Sensitivity, Specificity and Classification Performance for the 5-fold CV

‘*’ denotes the best CP for each CV scheme

(Sen:Sensitivity, Spec:Specificity and CP:Classification Performance)


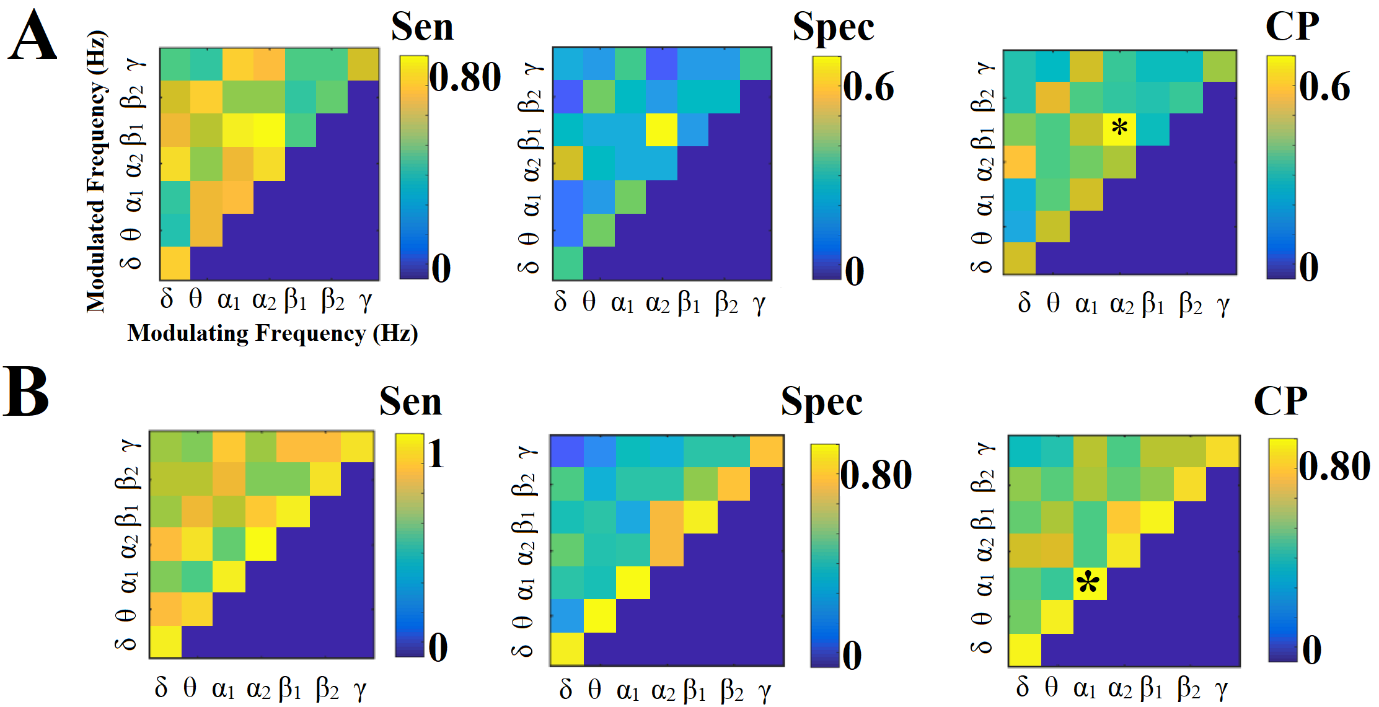


**S.Fig.2. Sensitivity, Specificity and Classification Performance of PLV using Centroid ROI representation and edge-weights approach of each SL-FCG.**

1. Sensitivity, Specificity and Classification Performance for the LOOCV and
2. Sensitivity, Specificity and Classification Performance for the 5-fold CV

‘*’ denotes the best CP for each CV scheme

(Sen:Sensitivity, Spec:Specificity and CP:Classification Performance)


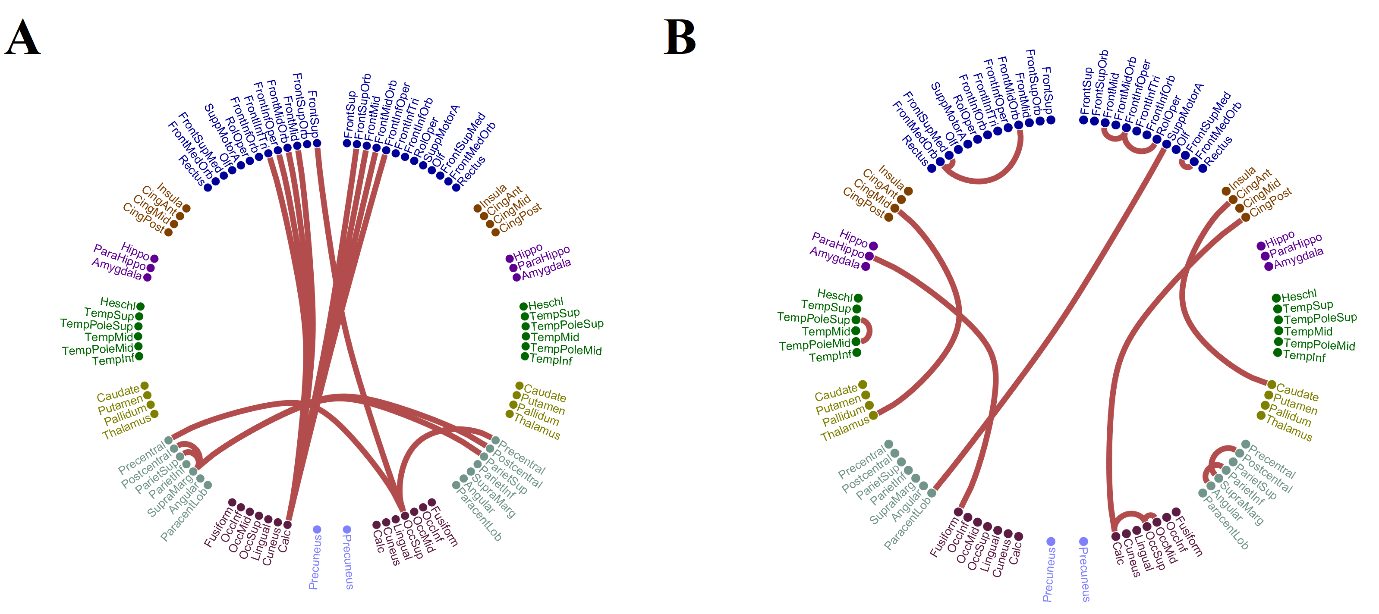


**S.Fig.3. Network topology of the selected edge-weighted features using the PLV connectivity estimator for** θ:α_2_**and** α_1_:α_1_ **intra-frequency coupling.**

The two network topologies differ on their ROI representation approach.

1. PCA ROI representation for θ:α_2_
2. Centroid ROI representation for α_1_:α_1_

The 90 ROI are illustrated circularly with 45 per hemisphere (left – right semi-circular distributions).

S.Fig. 3 illustrates the different network topology of the selected edge-weighted features in θ:α_2 /_ α_1_:α_1_ cross-frequency FCG based on both ROI representation schemes for the CorrEnv. PCA ROI approach reveal fronto-parietal connections with calcarine to be a major hub receiving connections from bilateral frontal areas (S.Fig.3.A). Centroid ROI scheme revealed connections within left and right frontal and interesting bilateral connections between parahippocampal and fusiform gyrus (S.Fig.3B).

**3.** **Classification Performance based on Edge –Weights in ML-FCG**

Following the same feature selection and cross-validation scheme in ML-FCG compared to SL-FCG, we extracted the 15 features highly consistent detected across the folds. S.Table 1 tabulates the sensitivity, specificity and classification performance of PLV connectivity estimator in both ROI representations. The classification performance was inferior for the PLV compared to iPLV reaching the 55% for the former compared to 87% for the latter which demonstrates again the superiority of the imaginary part of PLV.

**S.Table 1. Sensitivity, Specificity and Classification Performance of edge-weights in ML-FCG^PLV^ using the two different ROI representations (PCA and CENTroid) and two cross-validation schemes (Leave-one out cross validation and 5-fold)**

|  |  | **Sensitivity** | **Specificity** | **Classification Accuracy** |
| --- | --- | --- | --- | --- |
| **PCA** | **LOOCV** | 0.60 | 0.37 | 0.50 |
|  | **5-FOLD** | 0.56 $\pm$ 0.17 | 0.51 $\pm$ 0.26 | 0.53 $\pm$ 0.06 |
| **CENT** | **LOOCV** | 0.66 | 0.45 | 0.57 |
|  | **5-FOLD** | 0.73 $\pm$ 0.17 | 0.33$\pm$ 0.19 | 0.55 $\pm$ 0.14 |

4. **Classification Performance based on tensorial treatment of SL-FCG^iPLV^**

**S.**Fig. 4 and 5 illustrate the sensitivity, specificity and classification performance of CorrEnv using PCA and centroid ROI representation, correspondingly. Both ROI representations and CV schemes failed to demonstrate high classification performance in every **SL-FCG^PLV^.**


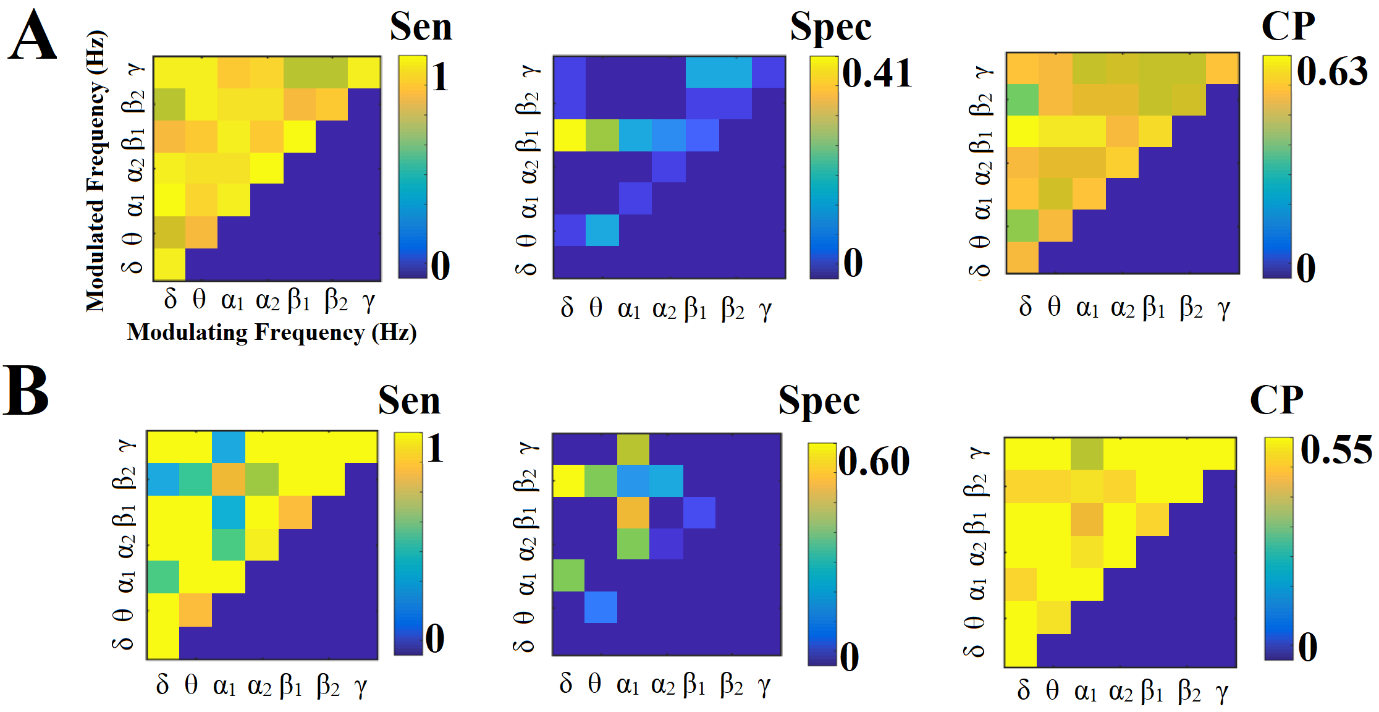


**S.Fig.4. Sensitivity, Specificity and Classification Performance of PLV using PCA ROI representation and tensorial treatment of each SL-FCG.**

1. Sensitivity, Specificity and Classification Performance for the LOOCV and
2. Sensitivity, Specificity and Classification Performance for the 5-fold CV

‘*’ denotes the best CP for each CV scheme

(Sen:Sensitivity, Spec:Specificity and CP:Classification Performance)


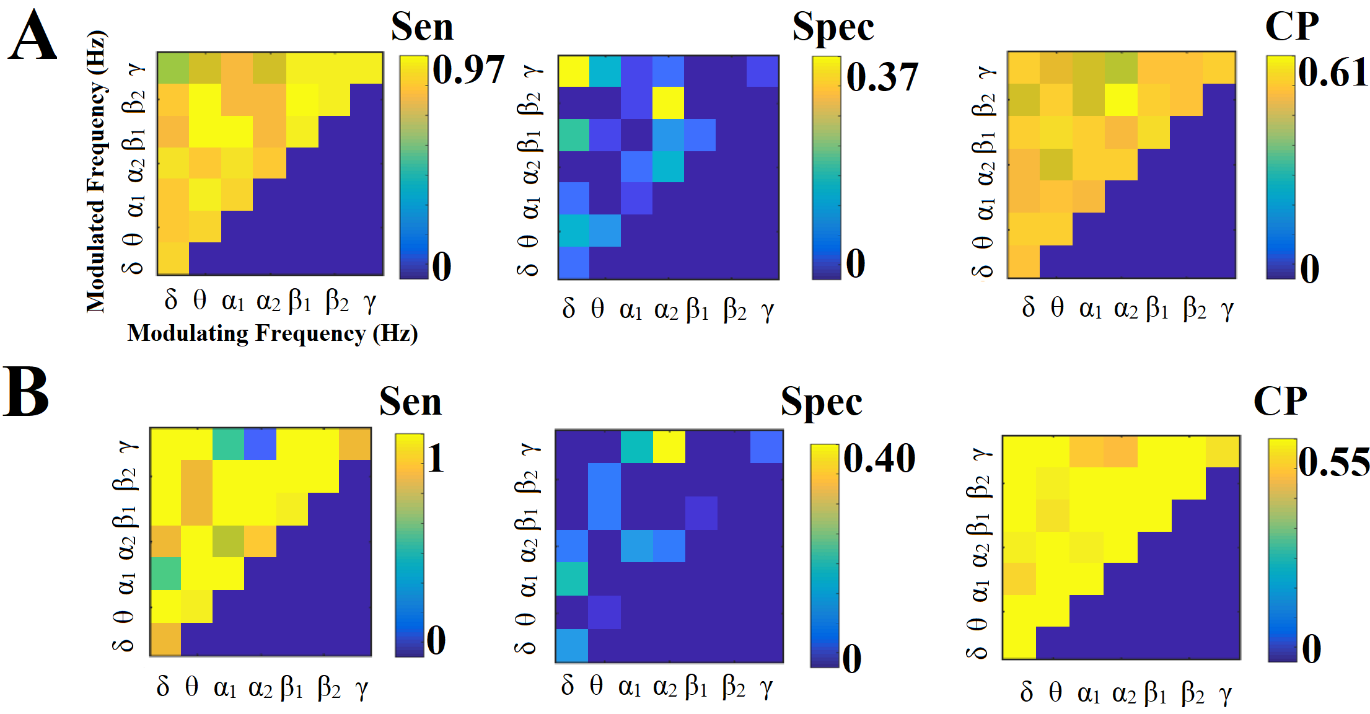


**S.Fig.5. Sensitivity, Specificity and Classification Performance of PLV using Centroid ROI representation and tensorial treatment of each SL-FCG.**

1. Sensitivity, Specificity and Classification Performance for the LOOCV and
2. Sensitivity, Specificity and Classification Performance for the 5-fold CV

‘*’ denotes the best CP for each CV scheme

(Sen:Sensitivity, Spec:Specificity and CP:Classification Performance)

5. **Classification performance based on the tensorial treatment of ML-FCG^OMST^**

We followed the same tensorial feature extraction and cross-validation scheme in ML-FCG as the ones used for each SL-FCG. In both cases, the classification performance were on the level of by chance (50%), which demonstrates the difficulty of merging the edge-weights features from SL-FCG to a ML-FCG. In both estimators (see Table 4 & 5), we succeeded to improve the classification performance compared to each SL-FCG using the tensorial treatment of the FCG but our results were too low compared to the edge-weights approach.

**S.Table 2. Sensitivity, Specificity and Classification Performance of the tensorial treatment of ML-FCG^PLV^ using two ROI representation and two cross-validation schemes.**

|  |  | **Sensitivity** | **Specificity** | **Classification Accuracy** |
| --- | --- | --- | --- | --- |
| **PCA** | **LOOCV** | 0.90 | 0.04 | 0.51 |
|  | **5-FOLD** | 1.00 $\pm$ 0.00 | 0.04 $\pm$ 0.08 | 0.57 $\pm$ 0.04 |
| **CENT** | **LOOCV** | 0.96 | 0.00 | 0.53 |
|  | **5-FOLD** | 1.00 $\pm$ 0.00 | 0.04$\pm$ 0.08 | 0.57 $\pm$ 0.04 |

**6. Network Analysis and Comodulograms of ML-FCG^OMST^**

**6.1 Network Analysis of the ML-FCG^OMST^**

We estimated MPC on the ML-FCG^OMST^ based on the degree of each node at every single layer. The performance based on PLV was lower compared to the CorrEnv with both ROI representation (PCA/CENT) reaching the 38% and 42% of accuracy, correspondingly (S.Table 3).

**S.Table 3. Sensitivity, Specificity and Classification Performance of MPL estimated over the ML-FCG^PLV^ using two ROI representation and two cross-validation schemes.**

|  |  | **Sensitivity** | **Specificity** | **Classification Accuracy** |
| --- | --- | --- | --- | --- |
| **PCA** | **LOOCV** | 0.43 | 0.25 | 0.35 |
|  | **5-FOLD** | 0.53 $\pm$ 0.19 | 0 .21 $\pm$ 0.02 | 0.38 $\pm$ 0.10 |
| **CENT** | **LOOCV** | 0.53 | 0.29 | 0.42 |
|  | **5-FOLD** | 0.53 $\pm$ 0.19 | 0.21$\pm$ 0.02 | 0.38 $\pm$ 0.10 |

**6.2 Comodulograms of the ML-FCG^OMST^**

S.Figure 5 illustrates the group-averaged comodulograms for PLV. Each 2D plots demonstrate the probability distribution of selected edges via the OMST filtering approach across the multi-layer. The in-diagonal cells in comodulograms keep the PD of the functional connections within each layer (intra-frequency coupling) while the off-diagonal cells keep the PD of the functional connections between the layers (cross-frequency couplings). Even though it is not clear from the color-coded, there are on average 8 connections between every pair of δ modulator with the rest of modulated frequencies in every case (ROI representations x connectivity estimators). In all cases (ROI representation x connectivity estimators) the basic modulating frequency that functions as a central hub of both intra and inter-frequency layers is δ brain rhythm (S.Fig.5). There are only a few connections from δ to the rest of modulated frequencies (1^st^ column) and for that reason the colour code doesn’t help to visualize them. PD ROI representation didn’t affect the contribution of intra/inter frequency-coupling modes in both CorrEnv and iPLV connectivity estimators.

**^
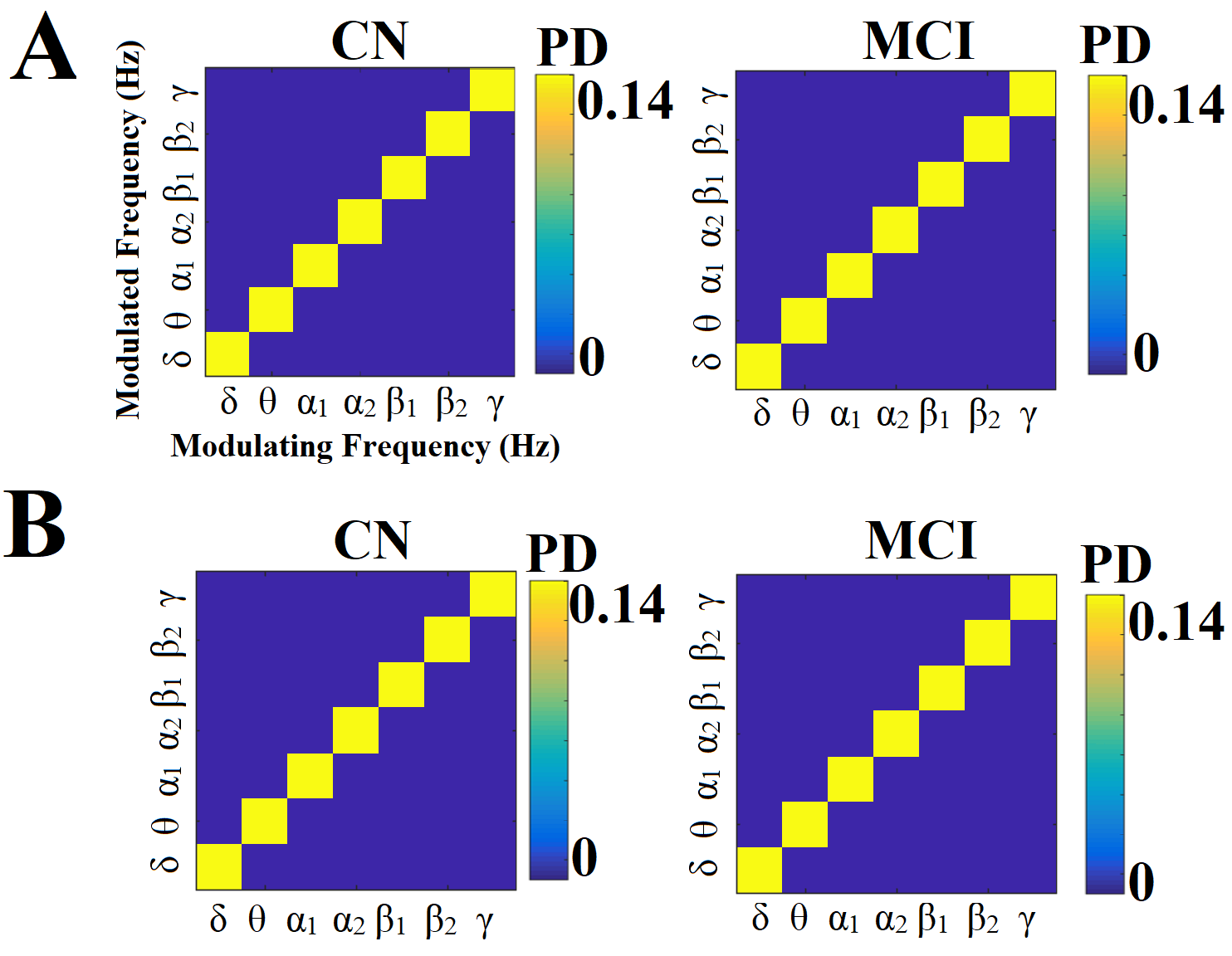
^**

**S.Fig.5. Group-averaged comodulograms derived from ML-FCG^PLV^.**

1. PCA ROI representation
2. Centroid ROI representation

(PD: Probability Distribution)
